# Development of the Caecal Microbiota in Three Broiler Breeds

**DOI:** 10.3389/fvets.2019.00201

**Published:** 2019-06-25

**Authors:** Peter Richards, Jo Fothergill, Marion Bernardeau, Paul Wigley

**Affiliations:** ^1^Institute of Infection and Global Health, University of Liverpool, Liverpool, United Kingdom; ^2^DuPont Industrial Biosciences, Genencor International BV, Leiden, Netherlands

**Keywords:** microbiota, chicks, broilers, Illumina, microbiology

## Abstract

The development of the caecal microbiota plays a role in the metabolism and immune competence of chickens. A detailed understanding of normal succession in the caecal microbiota can inform the use of probiotics and other interventions to optimize the caecal microbiota. The development of the microbiota in caecal mucus and lumen samples from three breeds of broiler chicken (Cobb 500, *n* = 36; Hubbard JA87, *n* = 38; and Ross 308, *n* = 36) was observed between 0 and 42 days post hatch. Chicks were housed in the same room of a climate-controlled, biosecure chicken housing unit. Between 0 and 14 days post hatch, chicks were kept in brooder pens ensuring a mixture of breeds in each brooder. From 22 days post hatch, chicks were removed from the brooders and kept in the same room. DNA was extracted from a pooled sample of caecal mucus and luminal contents from five birds of each breed at 0, 3, 7, 14, 21, 28, and 42 days post hatch. High-throughput Illumina sequencing was performed for the V4 hypervariable region of the 16S rRNA gene. The early caecal microbiota was characterized by poor diversity and dominance by one or two bacterial species. Early colonizers of the caecum included *Bifidobacteriaceae, Lachnospiraceae, Bacteroidaceae* and *Burkholderiaceae* with some amplicon sequence variants (ASVs) assigned to *Ruminococcaceae*. Later colonizers of the caecal microbiota were most apparent from 14 d.p.h and included *Ruminococcaceae, Clostridiales* vadin BB60 group, *Christensenellaceae* and *Bacillaceae*. The caecal microbiota continued to change until 42 d.p.h when the microbiota was characterized by a high abundance of *Bacteroidaceae, Lachnospiraceae* and *Ruminococcaceae*. The lumen microbiota was significantly different to the mucus with some ASVs assigned to *Lachnospiraceae, Ruminococcaceae, Christensenellaceae* and *Bacillaceae* showing increased abundance in the mucus. ASVs assigned to *Bacteroidaceae, Lactobacillaceae* and *Burkholderiaceae* showed a preference for the lumen. Analysis of five caecal mucus samples from each breed at 42 days post hatch showed differences in microbiota composition between Ross and Cobb as well as between Ross and Hubbard. Since performance data was not collected no functional inferences as to the significance of this finding can be made.

## 1. Introduction

The intestinal microbiota of an individual chicken may be composed of between 200 and 350 different bacterial species (1) while around 640 bacterial species have so far been identified in the chicken gastrointestinal tract (2). In recent years it has become apparent that this diverse range of bacteria are not innocuous bystanders but play a range of roles in the host, from metabolism to immune maturation (3). With this realization has come the desire to identify beneficial bacteria and modulate their abundance to accentuate their effects. However, before successful interventions can be implemented a more detailed understanding of the normal development of the intestinal microbiota is required. The caeca are often chosen as a site for microbiota investigation due to their importance in both metabolism and immune maturation. Digestive caecal processes, such as bacterial fermentation to produce short chain fatty acids, provide up to 10% of a chicken's metabolizable energy (4). In terms of importance in immunity, the caecum is an important site of colonization by pathogens such as *Campylobacter jejuni* as well as receiving constant bacterial challenge by environmental bacteria introduced by reflux from the urodeum and cloaca (5, 6).

Several studies have investigated the microbiota of day-old chicks, but when considering day-old chicks, there may be a problem with terminology. Day-old chicks sold by hatcheries may not be less than 24 h old and can be over 72 h old at point of sale. Microbes inhabiting the gut are derived from the environment during incubation, hatching and handling during delivery. This initial colonization is variable, and the composition of the initial intestinal microbiota will differ significantly between chicks from different hatcheries (7). This likely explains the wide variety of results obtained by different groups when examining the intestinal microbiota in day-old chicks. For example, Ballou et al. (8) identified *Gammaproteobacteria* as contributing roughly 85% of sequences recovered from caecal samples from day old chicks while Pedroso et al. (9) found that *Pelotomaculum*, a genus of *Clostridiales*, and *Enterococcus* were the most abundant genera at the same age.

Once chicks have arrived on the farm they are exposed to a more diverse microbial environment. Bacteria are ingested from litter, feed and water. Succession occurs rapidly and differentiation of populations in different gut compartments occurs at a young age. The caecal and ileal microbiota begin to diverge from about 3 days old (10, 11). Ballou et al. (8) describes the day-old chick microbiota as dominated by *Gammaproteobacteria*, mainly *Enterobacteriaceae*, with a smaller population of *Enterococcus*. By 3 days old, sequences belonging to *Ruminococcaceae* and other *Firmicutes* commonly found in the caecum, such as *Clostridiales*, are present. These were the dominant bacteria by 14 and 28 days old (8). Oakley et al. (12) found a caecal microbiota dominated by *Clostridiales* such as *Flavonifractor, Pseudoflavonifractor* and *Lachnospiraceae* by 7 days old. The microbiota remained dominated by *Clostridiales*, however the dominant genus switched to *Faecalibacterium* and *Roseburia* at 21 and 42 days old, respectively. Most importantly, the diversity of the caecal microbiota continued to develop, with a doubling of the number of genera between 7 and 42 days old (12). Wise and Siragusa (13) obtained similar results showing a shift from *Enterobacteriaceae* to *Clostridiales* by 14 days old. Zhu and Joerger (14), using FISH, describe a similar change in the caecal microbiota between 2 days and 6 weeks old with an increase in diversity. Initially, the microbial community was composed of *Enterobacteriaceae, Lactobacillus* and *Bifidobacterium*. These groups give way over time to produce a microbiota mainly composed of *Clostridiales* (14).

There is also some evidence that the intestinal microbiota can be influenced by host genotype in chickens (15–17). These studies have used divergent or inbred lines rather than commercial breeds making the results of limited direct use to the poultry industry. Another potential source of variation is that the experimental groups are often housed separately. Since environmental factors such as litter type and diet (18, 19) are known to affect the composition of the microbiota it is reasonable to question whether housing experimental groups separately introduces a confounding variable.

This study aims to revisit the topic of normal caecal microbiota development using the increased resolution of next generation sequencing to shed light on microbial succession. The second objective of this study was to observe the development of the caecal microbiota in three common breeds of broiler chicken (Cobb 500, Hubbard JA87, and Ross 308) whilst they are housed together.

## 2. Materials and Methods

### 2.1. Animals and Housing

One hundred and ten (36 Cobb 500, 38 Hubbard JA87 and 36 Ross 308) day-old chicks were obtained from a single commercial hatchery. Chicks were distributed across three circular brooder pens (2 metres diameter) in the same room of a climate-controlled, biosecure chicken housing unit. Each brooder used a wood shaving substrate and contained the same number of chicks from each breed. Chicks were tagged with colored wing tags to allow accurate identification of the different breeds but were not individually identifiable. Water and feed were provided *ad libitum* by a drinker and feeder in each brooder. Chicks were fed a pelleted vegetable protein-based starter diet (Special Diet Services, Witham, Essex, UK) until 14 days post hatch (d.p.h). From 14 d.p.h a pelleted vegetable protein-based grower diet (Special Diet Services, Witham, Essex, UK) was provided until the end of the experiment. Nutritional composition of the starter and grower diets is displayed in Table 1 with a full list of ingredients and additives provided in the Supplementary Table 1. No coccidiostats or antimicrobials were added to either diet due to the high biosecurity levels maintained in the housing. At 22 d.p.h the birds no longer required brooder lamps and, as such, they were removed from the brooders and housed together in the same room on wood shavings. Temperature in the birds pens was maintained between 25 and 30°C. No mortality was observed during the study. All experimental protocols were conducted in accordance with the Animals (Scientific Procedures) Act 1986 under project licence 40/3652 and was approved by the University of Liverpool Animal Welfare and Ethical Review Body prior to the award of the licence.

**Table 1:** Composition of starter and grower diets.

**Analytical constituents (%)**	**Diet**	
	**Starter**	**Grower**
Crude fat	2.7	2.4
Crude protein	18.9	15.6
Crude fiber	3.8	4.1
Crude ash	6.6	5.6
Lysine	0.99	0.69
Methionine	0.44	0.27
Calcium	1.05	0.89
Phosphorus	0.7	0.62
Sodium	0.15	0.15
Magnesium	0.17	0.22
Copper	15 mg/kg	16 mg/kg

### 2.2. Sample Collection

Five chickens of each breed were euthanised for sample collection at 0, 3, 7, 14, 21, 28, and 42 d.p.h giving a total of 15 birds sampled at each time point. After euthanasia by cervical dislocation the abdomen was sprayed with 70% ethanol. Skin incisions were made to expose the sternum which was then reflected to give good access to the coelom. One caecum was removed and the contents manually expressed into a sterile container. Any visible digesta remaining was manually expressed but the caecum was not rinsed before sampling the mucus layer. The caecum was opened longitudinally using a sterile scalpel which was then used to gently scrape off the mucus layer and transfer the mucus to a sterile container. Mucus samples were taken from 14 d.p.h as the caeca at earlier time points were too small to yield adequate mucus for accurate pooling. Samples from 0 and 3 d.p.h were weighed and pooled by breed as some chicks yielded less than 200 mg of content. For all other time points, 200mg of caecal content from each bird was taken and pooled by breed. Mucus samples were weighed, diluted with 500 μl of sterile water, pooled by breed and homogenized. At 42 d.p.h, the 5 samples of caecal mucus taken from each breed were pooled but also stored for individual sequencing to assess differences in the mature caecal microbiota between breeds. The pooled samples were flash frozen in liquid nitrogen and stored at −20° C for 5 weeks before DNA extraction.

### 2.3. DNA Extraction

DNA was extracted from each sample using Zymobiomics DNA MiniKits (Cambridge Bioscience, UK) according to the manufacturer's instructions. DNA was extracted from 200 mg of luminal content and 250 μl of homogenized mucus. An initial bead-beating step was performed using a Qiagen TissueLyser at 30 Hz for 10 min. DNA was extracted from samples serially to ensure that storage time was equal for each time point. At each extraction, two controls were included: a blank extraction to control for contamination and 75 μl of Zymobiomics Standard Bacterial Community (Cambridge Bioscience, UK) to control for variations in DNA extraction efficacy. Extracted DNA was quantified using a NanoDrop 2000 spectrophotometer (NanoDrop Technologies) and a Qubit dsDNA HS fluorometric kit (Invitrogen).

### 2.4. Illumina MiSeq Sequencing

Extracted DNA was sent for paired-end sequencing of the 16S rRNA gene at the Centre for Genomic Research (University of Liverpool) using an Illumina MiSeq run. The V4 hypervariable region (515F/R806) was amplified to yield an amplicon of 254 base pairs (20). Library preparation was performed using a universal tailed tag design with subsequent amplification performed using a two step PCR with a HiFi Hot Start polymerase (Kapa) (21). The first round of PCR was performed using the primers 5′-ACACTCTTTCCCTACACGACGCTCTTCCGATCTNNNNNGTGCCAGCMGCCGCGGTAA-3′ (forward) and 5′-GTGACTGGAGTTCAGACGTGTGCTCTTCCGATCTGGACTACHVGGGTWTCTAAT-3′ (21). The raw Fastq files were trimmed for the presence of Illumina adapter sequences using Cutadapt version 1.2.1. The reads were further trimmed using Sickle version 1.200 with a minimum window quality score of 20. Reads shorter than 10 base pairs after trimming were removed. Raw sequence reads are available in the NCBI Sequence Repository Archive under the accession number SRP158778.

### 2.5. Amplicon Sequence Variant Identification and Taxonomy Assignment

QIIME2 version 2018.4.0 was used for analysis of the Illumina data (22). Amplicon sequence variant (ASV) assignment was completed using the dada2 plugin (23) and a feature table produced using the feature-table plugin (https://github.com/qiime2/q2-feature-table) to produce a BIOM format table (24). The resulting feature table was divided into three individual tables: one containing all pooled samples to observe development of the caecal microbiome, one containing pooled samples from 14 d.p.h onwards to analyse differences between mucus and lumen microbiota and one containing individual samples taken at 42 d.p.h from Ross, Hubbard and Cobb chickens to assess differences in the microbiota between breeds. Taxonomy was assigned using the q2-feature-classifier plugin (25) with a pre-trained NaiveBayes classifier based on the SILVA 132 database of the 515F/R806 region of the 16S rRNA gene (26) available for download at https://docs.qiime2.org/2018.11/data-resources/.

### 2.6. Data Analysis and Statistics

Alpha and beta diversity analyses were performed at a sampling depth of 23,000 using the alignment (27), phylogeny (28) and diversity (https://github.com/qiime2/q2-diversity) plugins. Alpha diversity, a metric used to assess species richness, was measured using an observed ASVs metric and compared between samples using a Kruskal Wallis test with a false discovery rate (FDR) correction. Taxa plots were produced using the q2-taxa plugin (https://github.com/qiime2/q2-taxa). Beta diversity, a metric used to compare species diversity and abundance between samples, was calculated with a weighted UniFrac metric. The beta diversity matrix was used to draw principal coordinate analysis (PCoA) plots and an ANOSIM test was used to determine the significance of differences in beta diversity between groups.

Gneiss analysis was chosen to analyse differential abundance between groups since it overcomes challenges created by the compositional nature of microbiota data. To facilitate interpretation of results, less common taxa were filtered by excluding ASVs with less than the median frequency. Next, a dendrogram of ASVs is prepared using correlation clustering. Each node in the dendrogram is treated as a “balance” with taxa on one side of the balance termed numerators and on the other, denominators. Gneiss analysis examines the log ratio of abundances between numerator and denominator taxa at each balance. Each log ratio's final numerical value is dependent on the balance between the taxa composing the numerator and those composing the denominator of the ratio. Differences in the log ratio of a balance can be compared between sample groups to determine differences in microbiota composition. A significant difference between samples allows hypotheses to be formulated regarding changes in the absolute abundance of numerator and denominator taxa but gives no further information as to which hypothesis is correct. For example, if balance y0 is found to be significantly lower at Time A compared to Time B the following hypotheses could explain the result: (i) The numerator taxa have increased between times A and B; (ii) The denominator taxa have an decreased between times A and B; (iii) A combination of hypotheses (i) and (ii); (iv) Both numerator and denominator taxa have increased between times A and B, but numerator taxa have increased more; (v) Both numerator and denominator taxa have decreased between times A and B, but denominator taxa have decreased more. Further investigations, such as quantitative PCR, are required to discern which hypothesis is correct (29).

Gneiss analysis (29) was run using the gneiss plugin (https://biocore.github.io/gneiss/) to identify taxa which were differentially abundant between time points and area sampled. Principal balances for use in Gneiss were obtained via Ward's hierarchical clustering using the correlation-clustering command. Isometric log ratios for each balance were calculated using the ilr-transform command. A multivariate response linear regression model of log ratios balances was constructed with area, breed and days post hatch as covariates using the ols-regression command. Results were visualized through a regression summary and dendrogram heatmaps. Balances significantly affected by the covariates “days post hatch,” “breed,” and “area” were identified as those with a *p*-value less than 0.05. The results of this analysis were used to select taxa for further analysis using quantitative PCR.

### 2.7. Quantitative PCR

Taxa were selected for further exploration using quantitative PCR based on results from Gneiss analysis. A literature search was conducted to find suitable primers. Where suitable primers were not available, the sequences retrieved from Illumina sequencing were used to produce taxa specific primers. The sequence was input into Primer-BLAST and a suitable primer pair was chosen. To test specificity of primers, each primer pair was input into TestPrime for comparison against the SILVA database SSU-r132. Further testing of primers was conducted using PCR. The primers were tested against known positive and negative samples to check for the correct amplicon size and non-specific amplification. A gradient PCR was conducted to establish the correct annealing temperature for quantitative PCR. Primers used are displayed in Table 2.

**Table 2:** Primer pairs used for quantitative PCR.

**Target taxa**	**Primers**	**Amplicon size (b.p.)**	**References**
Domain *Bacteria* (targets V4 region)	F: TGCCAGCMGCCGCGGTAA	254	(30)
	R: GGACTACHVGGGTWTCTAAT		
*Bacteroides*	F: CCTWCGATGGATAGGGGTT	131	(31)
	R: CACGCTACTTGGCTGGTTCAG		
*Lachnospiraceae-Ruminococcaceae*	F: CGGTACCTGACTAAGAAGC	429	(32)
	R: AGTTTYATTCTTGCGAACG		
*Bacillus*	F: GCATTGGAAACTGGGGGACT	90	This study
	R: CCGGTGTTCCTCCACATCTC		
*Bifidobacterium*	F: CTCCTGGAAACGGGTGG	550	(33)
	R: GGTGTTCTTCCCGATATCTACA		
*Clostridium* cluster IV	F: TTACTGGGTGTAAAGGG	580	(34)
	R: TAGAGTGCTCTTGCGTA		
*Clostridium* cluster XIV a&b	F: AAATGACGGTACCTGACTAA	438-441	(35)
	R: CTTTGAGTTTCATTCTTGCGAA		

The real-time quantitative PCR assay was conducted on a 1:10 solution of extracted DNA with a Rotor-Gene Q (Qiagen) and Rotor-Gene SYBR Green PCR kits (Qiagen). The V4 region of the 16S rRNA gene was used as a reference gene. Rotor-Gene Q software (version 2.3.1.49) was used to produce melting curves and identify the cycle threshold (Ct), the point at which fluoresence above the background level is detectable. Each sample was run in triplicate with an averaged Ct used in further analysis. The ΔCt, defined as the difference between the Ct value for taxa specific primers and the Ct value for the reference gene, was calculated for each sample. Results were expressed as 40 −ΔCt. Results from qPCR were compared to the relative abundance of the corresponding taxonomic group using a Spearman rank-order correlation coefficient to assess correlation between abundance determined by qPCR and sequencing.

## 3. Results

### 3.1. Succession in the Caecal Microbiota

#### 3.1.1. Alpha and Beta Diversity

**Figure 1:**
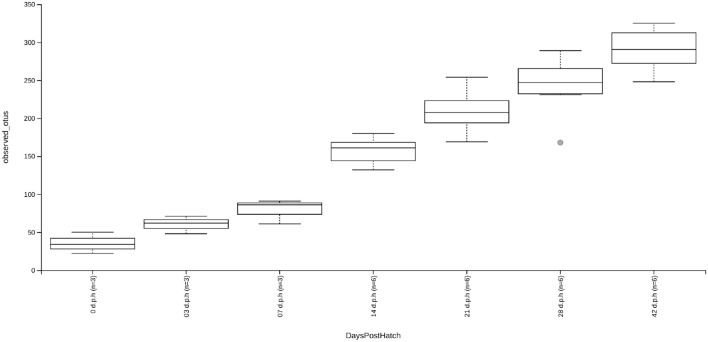
Box and whisker plots showing alpha diversity, as measured by an observed ASVs metric, for samples taken at 0, 3, 7, 14, 21, 28, and 42 d.p.h. Although increases in alpha diversity were observed between subsequent time points, significant increases in alpha diversity were found between 7 and 14 d.p.h, 14 and 21 d.p.h, and 28 and 42 d.p.h. The increases in alpha diversity between other time points was likely insignificant due to small sample size.

At hatch, the caecum was populated by an average of 35 ASVs. By 3 d.p.h, this increased to an average of 60 ASVs although this change was not significant likely due to the small sample size used. There was no significant change in alpha diversity between 3 and 7 d.p.h although the average number of ASVs increased to 79 nor was there a significant change between 21 and 28 d.p.h despite the average number of ASVs increasing from 209 to 241. There were significant increases in alpha diversity between 7 and 14 d.p.h (*H* = 5.4, *p* = 0.02), 14 and 21 d.p.h (*H* = 7.0, *p* = 0.02) and 28 and 42 d.p.h (*H* = 4.3, *p* = 0.04). At 42 d.p.h, there was an average of 290 ASVs present in the caecal microbiota (Figure 1).

**Figure 2:**
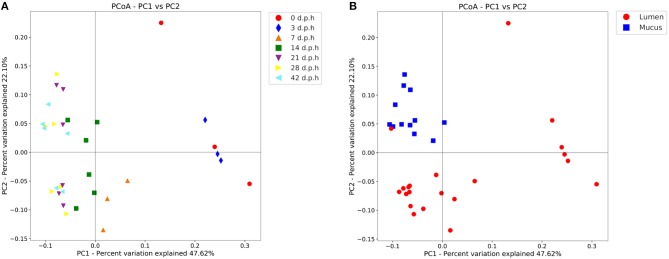
Principal coordinate analysis (PCoA) plot showing differences in weighted UniFrac beta diversity at different time points **(A)** and areas sampled **(B)**. Each point represents a pooled sample with distance between points representative of differences in microbiota composition. Between 0 and 14 days post hatch, the changes in beta diversity between time points are notable with large distances between samples taken at 0, 3, 7, and 14 d.p.h. There are no significant changes in beta diversity from 21 days post hatch which is evidenced by the closer clustering of samples from 21, 28, and 42 d.p.h. Mucus and lumen samples form separate clusters suggesting a different microbiota composition.

From the *β*-diversity plots a pattern of succession becomes apparent (Figure 2). When measured with a weighted UniFrac metric, beta diversity was significantly affected by days post hatch (R-statistic = 0.50, *p* = 0.001). The caecal community at 0 and 3 d.p.h was different when compared to other time points with more variation between samples taken at the same time. At 7 d.p.h the microbiota was more similar to later time points. Samples from 14 d.p.h continue to cluster separately, however, samples from 21 d.p.h cluster together with 28 and 42 d.p.h samples. There was also a noticeable separation between mucus and luminal samples from 14 d.p.h (Figure 2).

#### 3.1.2. Differential ASVs Between Time Points

**Figure 3:**
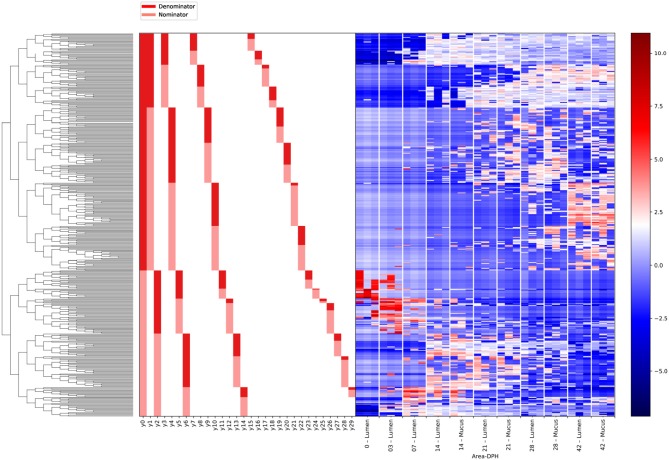
A dendrogram heatmap showing log abundance of ASVs in the caecal microbiota between 0 and 42 days post hatch. There is a visible pattern of colonization between 0 and 42 days post hatch over which time the microbiota becomes more diverse.

Gneiss analysis and taxa plots revealed differential ASV abundance between time points. The feature table was filtered to exclude ASVs with a frequency of less than 102, reducing the number of ASVs included from 847 to 424. For Gneiss analysis, the overall linear regression model fit was R2 = 0.654 with covariate “days post hatch” accounting for 56% of variance. Log ratio balances y0 (42 d.p.h: β = −31.1, *p* < 0.001; 28 d.p.h: β = −22.9, *p* < 0.001; 21 d.p.h: β = −10.7, *p* = 0.02), y1 (42 d.p.h: β = −20.4, *p* < 0.001; 28 d.p.h: β = −25.4, *p* < 0.001; 21 d.p.h: β = −25.7.5, *p* < 0.001; 14 d.p.h: β = −21.5, *p* < 0.001; 7 d.p.h: β = −7.1, *p* = 0.007), y2 (42 d.p.h: β = 21.5, *p* < 0.001; 28 d.p.h: β = 26.2.5, *p* < 0.001; 21 d.p.h: β = 31.1, *p* < 0.001; 14 d.p.h: β = 32.2, *p* < 0.001; 7 d.p.h: β = 19.6, *p* < 0.001), y3 (21 d.p.h: β = −11.6, *p* = 0.001; 14 d.p.h: β = −20.2, *p* < 0.001; 7 d.p.h: β = −10.7, *p* = 0.008), y4 (42 d.p.h: β = 15.4, *p* < 0.001; 21 d.p.h: β = −8.0, *p* = 0.005), y5 (42 d.p.h: β = 14.4, *p* < 0.001; 28 d.p.h: β = 13.2, *p* < 0.001; 21 d.p.h: β = 14.5, *p* < 0.001; 14 d.p.h: β = 16.3, *p* < 0.001; 7 d.p.h: β = 20.2, *p* < 0.001, 3 d.p.h: β = 22.9, *p* < 0.001), y6 (42 d.p.h: β = 8.8, *p* < 0.001; 14 d.p.h: β = 6.5, *p* = 0.01; 7 d.p.h: β = 21.7, *p* < 0.001, 3 d.p.h: β = 9.6, *p* = 0.001), y7 (14 d.p.h: β = 5.3, *p* = 0.006; 7 d.p.h: β = 13.4, *p* < 0.001) and y8 (21 d.p.h: β = 11.9, *p* < 0.001; 14 d.p.h: β = 7.7, *p* = 0.04) were significantly different at one or more time points. On inspection of the dendrogram heatmap (Figure 3) these balances revealed waves of colonization in the caecal microbiota. The taxonomic assignments at the level of family of ASVs in these balances are displayed in Table 3 and full taxonomy plots are provided in the Supplementary Material.

**Table 3:** Taxonomy of ASVs present in balances which reveal significant differences between time points.

**Time of colonization**	**Balances of interest**	**Family**	**Number of ASVs**
0 days post hatch	y5_denominator_	*Clostridiaceae* 1	14
		*Enterobacteriaceae*	12
		*Enterococcaceae*	3
		*Peptostreptococcaceae/Lachnospiraceae*	1
3 days post hatch	y5_numerator_	*Lachnospiraceae*	29
	y6_numerator_	*Ruminococcaceae*	10
		*Enterococcaceae*	6
		*Clostridiaceae* 1	4
		*Enterobacteriaceae/Eggerthellaceae/*	
		*Burkholderiaceae/Lactobacillaceae*	3
		*Peptostreptococcaceae/Erysipelotrichaceae/*	
		*Coriobacteriaceae/Eubacteriaceae*	2
		*Peptostreptococcaceae/Lactobacillaceae/*	
		*Bifidobacteriaceae*	1
7 days post hatch	y7_numerator_	*Lachnospiraceae*	9
		*Ruminococcaceae*	4
		*Bacteroidaceae*	2
		*Atopobiaceae*	1
14 days post hatch	y6_denominator_	*Ruminococcaceae*	53
	y7_denominator_	*Lachnospiraceae*	38
	y8_numerator_	*Clostridiales* vadin BB60 group	3
		*Lactobacillaceae*	2
		*Staphylococcaceae/Bacillaceae/*	
		*Peptostreptococcaceae/Burkholderiaceae/*	
		*Christensenellaceae*	1
21 days post hatch	y8_denominator_	*Ruminococcaceae*	48
	y4_denominator_	*Lachnospiraceae*	28
		*Clostridiales* vadin BB60 group	9
		*Christensenellaceae*	6
		*Bacillaceae*	4
		*Mollicutes* RF39 */Erysipelotrichaceae*	3
		*Eggerthellaceae*	2
		*Peptococcaceae/Staphylococcaceae/*	
		*Clostridiaceae* 1 */Bacteroidaceae*	1
28 and 42 days post hatch	y4_numerator_	*Ruminococcaceae*	48
		*Clostridiales* vadin BB60 group	17
		*Lachnospiraceae*	16
		*Peptococcaceae*	3
		*Lactobacillaceae/Christensenellaceae/*	
		*Clostridiales*	2
		*Erysipelotrichaceae/Defluviitaleaceae/*	
		*Atopobiaceae/Clostridiales* Family XIII/	
		*Coriobacteriaceae*	1

*Taxonomies of individual balances are provided in Supplementary Figure 1*.

**Figure 4:**
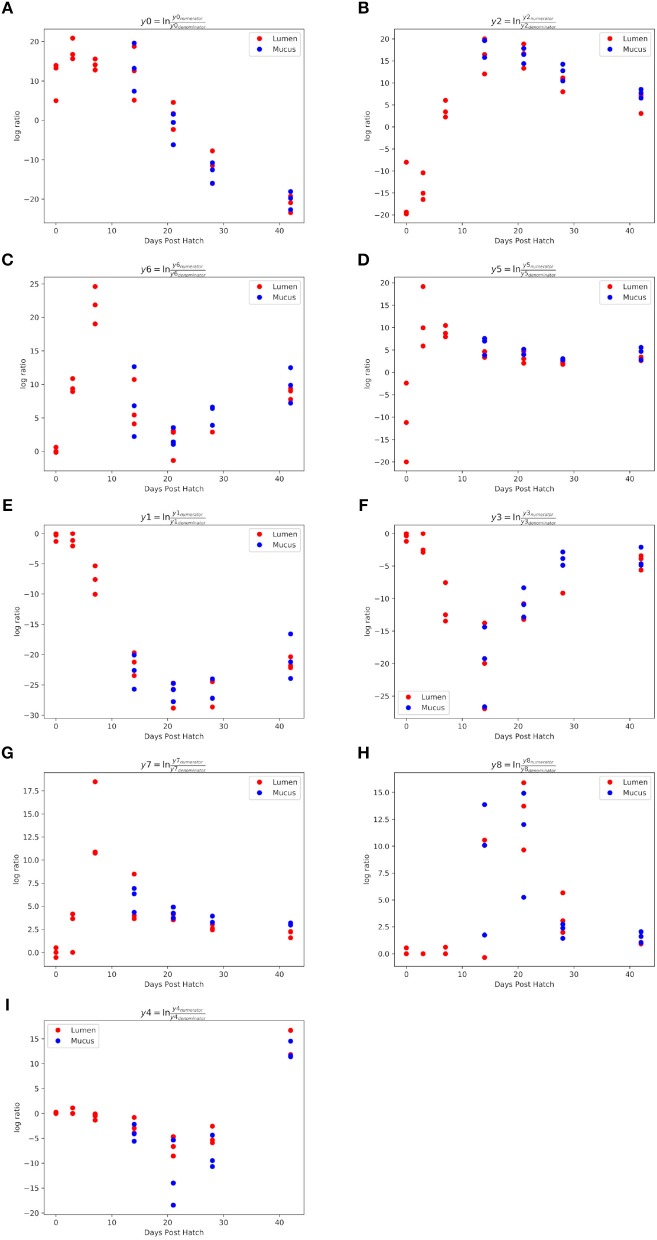
Log ratios of balances significantly different between 0 and 42 d.p.h. Balances y0 **(A)**, y2 **(B)**, y6 **(C)**, y5 **(D)**, y1 **(E)**, y3 **(F)**, y7 **(G)**, y8 **(H)** and y4 **(I)** were identified by Gneiss analysis as significantly different between time points. A lower log ratio shows a shift in the balance toward denominator ASVs whilst a higher log ratio shows a shift toward numerator ASVs. The balances shown above describe stages of colonization observed in the study.

**Figure 5:**
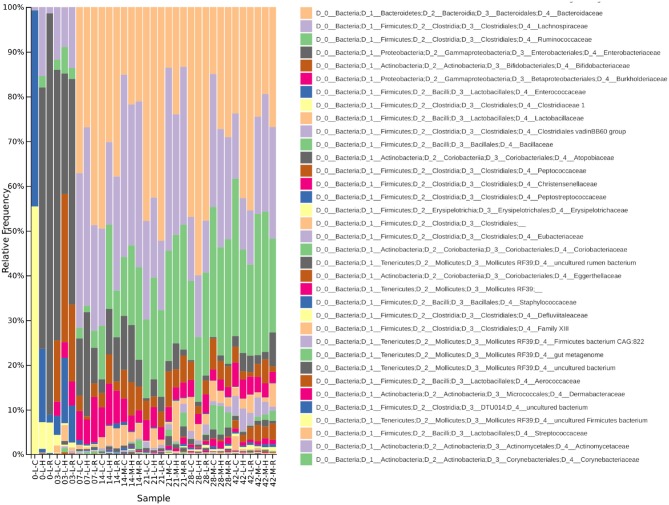
A taxa plot showing relative abundance of bacterial Families within the chicken caecal microbiota. Samples from the lumen (L) and mucus (M) of Cobb (C), Hubbard (H), and Ross (R) broilers are shown between 0 and 42 d.p.h.

The log ratio of balance y0 had a high value at 0 d.p.h suggesting that some ASVs in y0_numerator_ were more abundant at this time (Figure 4A). Between 3 and 42 d.p.h, the log ratio decreased as there was a shift in relative abundance from y0_numerator_ to y0_denominator_ ASVs. The initial abundance and subsequent decline of y0_numerator_ ASVs can be observed in the dendrogram heatmap (Figure 3) and the taxa plot (Figure 5). However, examination of further balances is required to fully distinguish ASVs prevalent at 0 d.p.h from those present at 3, 7, and 14 d.p.h.

Balance y2 is a subdivision of y0_numerator_. The log ratio of balance y2 was lower at 0 d.p.h as there was a higher abundance of y2_denominator_ ASVs (Figure 4B). There was an increase in log ratio from 7 to 14 d.p.h as the relative abundance of numerator ASVs increased (Figure 3). From 21 d.p.h, the log ratio began to decrease as the balance was shifted back toward 0 by a decrease in relative abundance of y2_numerator_ ASVs. It can be concluded that ASVs which colonized the caecum between 7 and 14 d.p.h are represented by y2_numerator_. Further resolution is provided by balance y6 whose log ratio increased from 0 to 7 d.p.h due to an increase in y6_numerator_ ASVs at 3 and 7 d.p.h before declining at 14 d.p.h as y6_denominator_ ASVs began to colonize the caecum (Figure 4C).

Balance y5 is a subdivision of y2_denominator_ which allows the distinction between ASVs which colonized at 0 and 3 d.p.h. The log ratio of balance y5 increased from 0 to 3 d.p.h (Figure 4D) suggesting that y5_denominator_ ASVs were more abundant at 0 d.p.h with a higher prevalence of y5_numerator_ ASVs at 3 d.p.h. This pattern is confirmed by the dendrogram heatmap (Figure 3).

As previously discussed, ASVs associated with later time points are described by y0_denominator_, but further balances are required to discern at which time point ASVs became prevalent. Balance y1 is a subdivision of y0_denominator_. The log ratio of balance y1 was close to 0 at 0 and 3 d.p.h as both numerator and denominator ASVs were absent. There was a slight decrease at 7 d.p.h as some denominator ASVs colonized the caecum. This decrease continued at 14 and 21 d.p.h before the log ratio began to increase again at 42 d.p.h (Figure 4E). This suggests that y1_denominator_ ASVs were associated with colonization between 7 and 21 d.p.h while y1_numerator_ ASVs represent later colonizers.

Balance y3 is a subdivision of y1_denominator_. The log ratio of balance y3 was close to 0 at 0 and 3 d.p.h as discussed for balance y1. There was a decrease in log ratio between 7 and 14 d.p.h followed by an increase from 21 d.p.h (Figure 4F). This shows that y3_denominator_ ASVs were associated with colonization between 7 and 14 d.p.h while y3_numerator_ ASVs represent later colonizers. Balance y7 provides further resolution of y3_denominator_ ASVs and shows that y7_numerator_ ASVs were associated with colonization at 7 d.p.h while y7_denominator_ ASVs began to colonize the caecum from 14 d.p.h (Figure 4G). Equally, balance y8 provides further resolution of y3_numerator_ ASVs and shows that y8_numerator_ ASVs colonized the caecum at 14 d.p.h while y8_denominator_ colonized from 21 d.p.h (Figure 4H).

Finally, balance y4 is a subdivision of y1_numerator_. The log ratio of balance y4 was close to 0 at 0, 3 and 7 d.p.h. There was a slight decrease in log ratio at 14 d.p.h with a further decrease noticeable at 21 d.p.h (Figure 4I). The dendrogram heatmap shows that this decrease in log ratio can be attributed to an increase in the relative abundance of y4_denominator_ ASVs. There was an increase in log ratio at 28 and 42 d.p.h attributable to an increase in the relative abundance of y4_numerator_ ASVs.

In conjunction with the taxa plot (Figure 5), these results demonstrate a pattern of succession within the caecum. ASVs identified as more abundant at 0 d.p.h were assigned to *Clostridiaceae* 1 (*n* = 14), *Enterobacteriaceae* (*n* = 12) and *Enterococcaceae* (*n* = 3). This pattern is visible in the taxa plot where these families are prevalent at 0 d.p.h. One ASV assigned to *Lachnospiraceae* was differentially abundant at 0 d.p.h. The taxaplot shows a small percentage of *Lachnospiraceae* and *Ruminococcaceae* detected in the microbiota at this early time point. Additionally, one ASV assigned to *Peptostreptococcaceae* was differentially abundant at 0 d.p.h.

The majority of ASVs identified as differentially abundant at 3 d.p.h were assigned to *Lachnospiraceae* (*n* = 29), *Ruminococcaceae* (*n* = 10) and *Enterococcaceae* (*n* = 6). Fewer ASVs abundant at 3 d.p.h were assigned to *Clostridiaceae* 1 (*n* = 4) and *Enterobacteriaceae* (*n* = 3). Other taxa which went on to form a significant part of the caecal microbiota were first abundant at this time point including *Lactobacillaceae* (*n* = 3), *Burkholderiaceae* (*n* = 3), *Eggerthellaceae* (*n* = 3), *Peptostreptococcaceae* (*n* = 2), *Eubacteriaceae* (*n* = 2), *Coriobacteriaceae* (*n* = 2) and *Bifidobacteriaceae* (*n* = 1).

Fewer ASVs were differentially abundant at 7 d.p.h but, again, the majority were assigned to *Lachnospiraceae* (*n* = 9) and *Ruminococcaceae* (*n* = 4). Two ASVs assigned to *Bacteroidaceae*, a major constituent of the caecal microbiota in this experiment, were first seen colonizing the caecum at 7 d.p.h. One ASV assigned to *Atopobiaceae* was differentially abundant at 7 d.p.h.

Most ASVs identified as differentially abundant at 14 d.p.h were assigned to *Ruminococcaceae* (*n* = 53) and *Lachnospiraceae* (*n* = 38). Several taxonomic groups colonized the caecum for the fist time at 14 d.p.h including *Clostridiales* vadin BB60 group (*n* = 3), *Staphylococcaceae* (*n* = 1), *Bacillaceae* (*n* = 1) and *Christensenellaceae* (*n* = 1). Two ASVs assigned to *Lactobacillaceae* and one assigned to *Burkholderiaceae* were differentially abundant at 14 d.p.h.

A similar pattern of colonization is shown by differentially abundant ASVs at 21 d.p.h with *Ruminococcaceae* (*n* = 48) the most common taxonomic assignment followed by *Lachnospiraceae* (*n* = 28). Furthermore, taxa which first colonized at 14 d.p.h were represented to a greater extent including *Clostridiales* vadin BB60 group (*n* = 9), *Christensenellaceae* (*n* = 6) and *Bacillaceae* (*n* = 4). *Mollicutes* RF39 (*n* = 3) and *Peptococcaceae* (*n* = 1) represented new colonizers at 21 d.p.h. Other ASVs differentially abundant at 21 d.p.h were assigned to *Erysipelotrichaceae* (*n* = 3), *Eggerthellaceae* (*n* = 2), *Staphylococcaceae* (*n* = 1), *Clostridiaceae* 1 (*n* = 1) and *Bacteroidaceae* (*n* = 1).

Between 28 and 42 d.p.h, the majority of ASVs identified as differentially abundant were assigned to *Ruminococcaceae* (*n* = 48), *Clostridiales* vadin BB60 group (*n* = 17) and *Lachnospiraceae* (*n* = 16). *Peptococcaceae* (*n* = 3), first colonizing the caecum at 21 d.p.h, was also differentially abundant at these time points. Other ASVs identified as differentially abundant at 28 and 42 d.p.h included *Lactobacillaceae* (*n* = 2), *Christensenellaceae* (*n* = 2), *Clostridiales* (*n* = 2), *Erysipelotrichaceae* (*n* = 1), *Defluviitaleaceae* (*n* = 1), *Atopobiaceae* (*n* = 1), *Clostridiales* Family XIII (*n* = 1) and *Coriobacteriaceae* (*n* = 1).

### 3.2. Differences Between the Luminal and Mucosal-Associated Microbiota

Samples from 21, 28, and 42 d.p.h were used to compare lumen and mucus samples from a mature caecal microbiota. Samples from 14 d.p.h were excluded as there were significant differences in alpha diversity between 14 and 42 d.p.h suggesting that the microbiota was not fully developed at 14 d.p.h. This is confirmed by analysis of beta diversity as time post hatch had a small but significant effect on beta diversity when samples from 14 d.p.h were included in the analysis (R-statistic = 0.17, *p* = 0.041).

#### 3.2.1. Alpha and Beta Diversity

There were no significant differences in alpha diversity between lumen and mucus samples. However, there were significant differences in beta diversity. Mucus and luminal samples formed distinct clusters on a PCoA plot (Figure 2), although there is one exception to this pattern. The lumen sample which clustered with the mucus samples is from Cobb at 42 d.p.h. With reference to the taxa plots (Figure 5), it's clear that the composition of this sample was very similar to mucus taken from Cobb at 42 d.p.h. An ANOSIM test (R-statistic = 0.79, *p* = 0.001) showed that area sampled had a significant effect on beta diversity.

#### 3.2.2. Differential ASVs Between the Lumen and Mucus Microbiota

**Figure 6:**
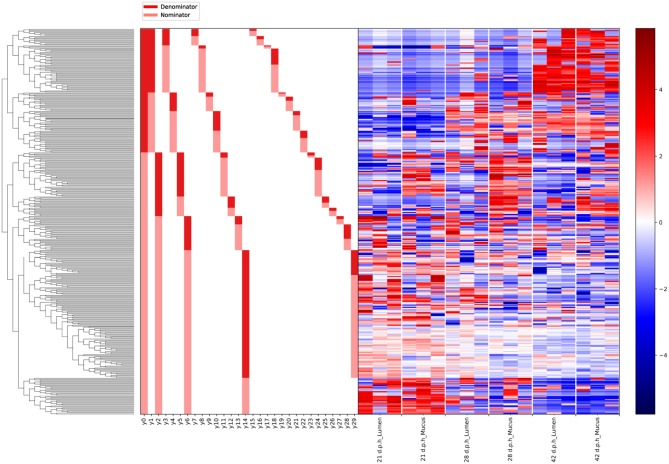
A dendrogram heatmap showing the log abundance of ASVs in the mucus and lumen. Differences in relative abundance between the lumen and mucus are visible in balance y2, identified by Gneiss analysis as containing differentially abundant ASVs.

Gneiss analysis (Figure 6 and Table 4) and taxa plots (Figure 5) revealed differential ASV abundance between mucus and lumen communities. The feature table was filtered to exclude ASVs with a frequency of less than 81, reducing the number of ASVs included from 748 to 375. The overall linear regression model fit was R2 = 0.56 with covariate “area” accounting for 7.9% of variance. Log ratio balance y2 (*β* = −11.5, *p* < 0.001) was a significant predictor for the covariate of “Area.”

**Table 4:** Taxonomy of ASVs contributing to the balance significantly affected by area sampled.

**Preferred niche**	**Balance of interest**	**Family**	**Number of ASVs**
Mucus	y2_denominator_	*Ruminococcaceae*	25
		*Lachnospiraceae*	20
		*Christensenellaceae*	5
		*Bacillaceae*	4
		*Eggerthellaceae*	2
		*Enterococcaceae/Erysipelotrichaceae/*	
		*Peptococcaceae/Coriobacteriaceae/*	
		*Peptostreptococcaceae/Mollicutes* RF39	1
Lumen	y2_numerator_	*Ruminococcaceae*	83
		*Lachnospiraceae*	75
		*Clostridiales* vadin BB60 group	9
		*Lactobacillaceae*	4
		*Burkholderiaceae*	4
		*Mollicutes* RF39	3
		*Bacteroidaceae*	3
		*Enterobacteriaceae*	2
		*Defluviitaleaceae/Peptostreptococcaceae/*	
		*Staphylococcaceae/Enterococcaceae/*	
		*Eubacteriaceae/Erysipelotrichaceae/*	
		*Christensenellaceae/Clostridiaceae/* 1	
		*Eggerthellaceae/Bifidobacteriaceae/*	
		*Atopobiaceae*	1

*Full taxonomies of significant balances are provided in Supplementary Figure 2*.

**Figure 7:**
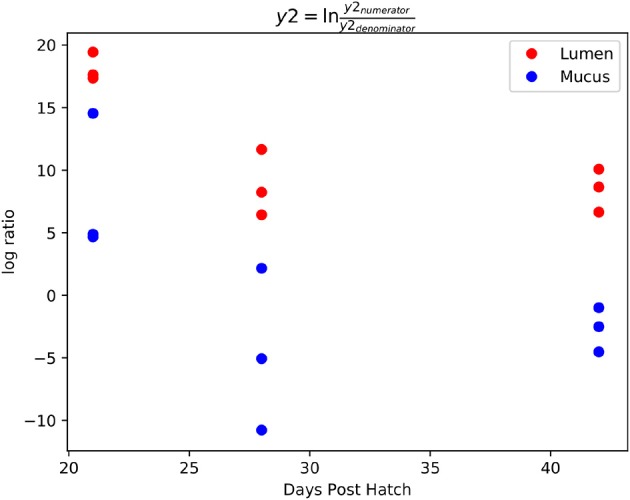
Log ratios of balance y2 which was significantly different between lumen and mucus samples. The lower log ratio in mucus samples suggests that the relative abundance of y2_denominator_ ASVs was higher in mucus samples compared to lumen samples.

The log ratio of balance y2 was lower in mucus samples compared to lumen samples showing that the relative abundance of y2_denominator_ ASVs was higher in the mucus than the lumen (Figure 7) with this increased abundance visible in the dendrogram heatmap (Figure 6). ASVs identified as more abundant in mucus were assigned to *Ruminococcaceae* (*n* = 25), *Lachnospiraceae* (*n* = 20), *Christensenellaceae* (*n* = 5) and *Bacillaceae* (*n* = 4). Other ASVs were assigned to *Eggerthellaceae* (*n* = 2), *Enterococcaceae* (*n* = 1), *Erysipelotrichaceae* (*n* = 1), *Peptococcaceae* (*n* = 1), *Coriobacteriaceae* (*n* = 1), *Peptostreptococcaceae* (*n* = 1) and *Mollicutes* RF39 (*n* = 1).

ASVs constituting y2_numerator_ were mostly assigned to *Ruminococcaceae* (*n* = 83), *Lachnospiraceae* (*n* = 75), *Clostridiales* vadin BB60 group (*n* = 9), *Lactobacillaceae* (*n* = 4) and *Burkholderiaceae* (*n* = 4). Other ASVs were assigned to *Mollicutes* RF39 (*n* = 3), *Bacteroidaceae* (*n* = 3), *Enterobacteriaceae* (*n* = 2) and one ASV to each of *Defluviitaleaceae, Peptostreptococcaceae, Staphylococcaceae, Enterococcaceae, Eubacteriaceae, Erysipelotrichaceae, Christensenellaceae, Clostridiaceae* 1, *Eggerthellaceae, Bifidobacteriaceae*, and *Atopobiaceae*.

The dendrogram heatmap does not show a pattern of increased relative abundance of y2_numerator_ ASVs in lumen samples compared to mucus samples. As a result, it is difficult to discern which y2_numerator_ ASVs are truly more abundant in the lumen as no other balances are significantly different allowing for greater resolution. However, the taxa plot can aid in this distinction. A paired Student's t-test of relative abundance of *Burkholderiaceae* (lumen average = 3.97%, mucus average = 2.8%, test statistic = 3.78, *p* = 0.005) and *Bacteroidaceae* (lumen average = 45.4%, mucus average = 21.4%, test statistic = 5.97, *p* < 0.001) show significant differences in relative abundance between lumen and mucus samples. On the other hand, some taxa in y2_numerator_ show a significantly higher relative abundance in mucus including *Bifidobacteriaceae* (lumen average = 2.8%, mucus average = 4.8%, test statistic = 4.11, *p* = 0.003), *Atopobiaceae* (lumen average = 0.68%, mucus average = 1.53%, test statistic = 4.04, *p* = 0.003) and *Eubacteriaceae* (lumen average = 0.02%, mucus average = 0.06%, test statistic = 3.11, *p* = 0.01). It's possible that since these taxa are represented by one or two ASVs that the limited sample size doesn't provide sufficient statistical power to demonstrate a significant difference in their abundance between mucus and lumen samples using Gneiss analysis. As a result, there is some doubt as to whether these taxa are differentially abundant between the mucus and the lumen.

#### 3.2.3. Differences Between the Lumen and Mucus Microbiota Confirmed by Quantitative PCR

Based on the results from Gneiss analysis and the taxa plot, quantitative PCR assays for *Bacteroidaceae, Lachnospiraceae*-*Ruminococcaceae, Bifidobacteriaceae* and *Bacillaceae* were performed. A combined primer pair for *Lachnospiraceae* and *Ruminococcaceae* was used as well as specific primers for *Clostridium* cluster XIVa&b and *Clostridium* cluster IV with the former corresponding roughly to *Lachnospiraceae* and the latter to *Ruminococcaceae*. Previously published primers were used for *Bacteroides* and *Bifidobacterium* detection as well-trialed primer pairs were available in the literature. The primers for the *Bacillaceae* ASVs were generated as described in the Materials and Methods section.

**Figure 8:**
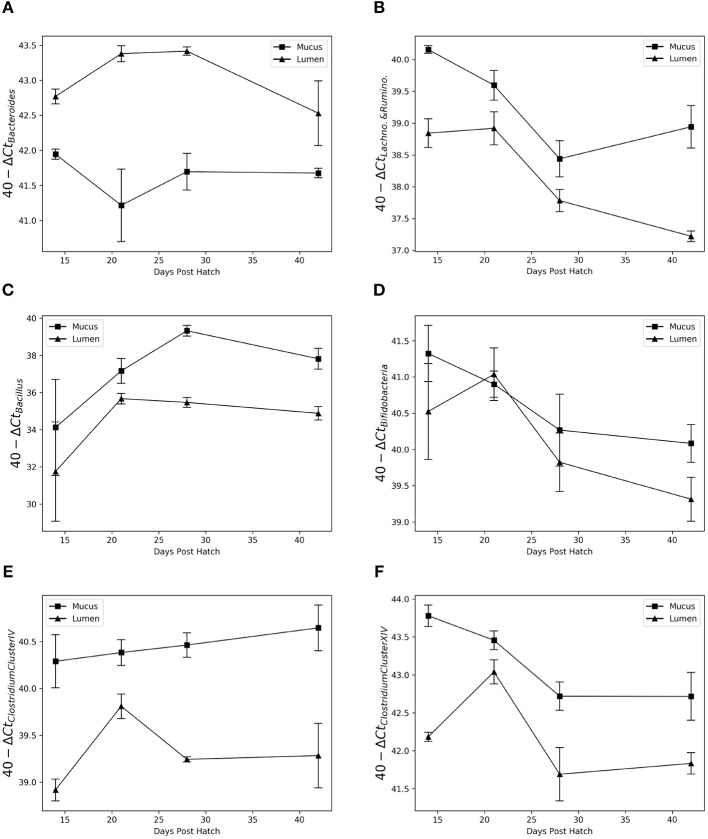
Relative abundance of *Bacteroides* **(A)**, *Lachnospiraceae-Ruminococcaceae* **(B)**, *Bacillus* **(C)**, *Bifidobacteria* **(D)**, *Clostridium* Cluster IV **(E)**, and *Clostridium* Cluster XIVa&b **(F)** in the mucus and lumen microbiota between 14 and 42 days post hatch. Data from different breeds was combined. Bacteroides shows a high affinity for the lumen while Bacillus is found almost exclusively in the mucus. A non-specific Lachnospiraceae-Ruminococcaceae primer pair showed consistently higher abundance in the mucus as do the more specific primers for *Clostridium* Cluster IV and XIVa&b. Bifidobacterium appears more abundant in the mucus; however this is not consistent across time points.

Results showed the same pattern as detected by Gneiss analysis (Figure 8). *Bacteroidaceae* were more abundant in luminal samples (test statistic = 6.3, *p* < 0.001) and *Bacillaceae* (test statistic = −2.4, *p* = 0.03) were more abundant in the mucus. *Bifidobacteriaceae* were more significantly abundant in the mucus, although this pattern breaks down at 21 d.p.h (test statistic = −2.5, *p* = 0.03). There was a significantly higher abundance of *Lachnospiraceae*-*Ruminococcaceae, Clostridium* cluster IV and *Clostridium* cluster XIVa&b in the mucus compared to the lumen (test statistic = −6.7, *p* < 0.001, test statistic = −9.1, *p* < 0.001 and test statistic = −5.8, *p* < 0.001, respectively).

The correlation between abundance determined by qPCR and relative abundance determined by sequencing varied between primers. There was a strong, positive correlation between results from qPCR and relative abundance determined by sequencing for *Bacillus* (rs_(22)_ = 0.89, *p* < 0.001), *Bacteroidaceae* (rs_(22)_ = 0.88, *p* < 0.001), *Clostridium* cluster XIVa&b (rs_(22)_ = 0.80, *p* < 0.001) and *Bifidobacteriaceae* [rs_(22)_ = 0.72, *p* < 0.001]. A weaker positive correlation was found between results from qPCR and relative abundance determined by sequencing for *Clostridium* cluster IV [rs_(22)_ = 0.49, *p* = 0.02] and *Lachnospiraceae*-*Ruminococcaceae* [rs_(22)_ = 0.40, *p* = 0.05].

### 3.3. Differences Between Breeds

The taxa plots of pooled samples showed some early differences in the caecal microbiota between breeds (Figure 5). At 0 d.p.h, there were large differences in composition between the breeds with Hubbard and Ross mainly colonized by *Enterobacteriaceae* while Cobb were colonized by *Enterococcaceae* and *Clostridiaceae* (Figure 5). By 3 d.p.h, there was increased homogeneity between the breeds although Hubbard had proportionally more *Bifidobacteriaceae* and less *Enterobacteriaceae* than the other two breeds. From 7 d.p.h, there were no visible differences between breeds as seen in the taxa plots.

#### 3.3.1. Alpha and Beta Diversity

Using data from 5 samples of caecal mucus from each breed taken at 42 d.p.h, some differences were noted. There was a significant difference in alpha diversity between Cobb and Ross samples (*H* = 4.84, *p* = 0.04) and Hubbard and Ross samples (*H* = 4.84, *p* = 0.04) but no significant difference between Cobb and Hubbard samples. Breed had a significant effect on beta diversity (R-statistic = 0.25, *p* = 0.03). Pair-wise tests between breeds revealed that there were no significant differences in beta diversity between Cobb and Hubbard samples and Ross and Hubbard samples. There was a significant difference between Cobb and Ross (ANOSIM: R-statistic = 0.57, *p* = 0.009).

#### 3.3.2. Differential ASVs Between Breeds

The feature table was filtered to exclude ASVs with a frequency of less than 66, reducing the number of ASVs included from 790 to 398. Pairwise Gneiss analysis between breeds revealed differential ASV abundance between caecal mucus microbiota in Cobb and Ross (Table 5) and Hubbard and Ross but found no appreciable difference between Hubbard and Cobb.

**Table 5:** Taxonomy of ASVs contributing to balances significantly affected by breed in a comparison between caecal mucus samples taken at 42 d.p.h from Cobb and Ross.

**Preferred breed**	**Balance of interest**	**Family**	**Number of ASVs**
Ross	y2_denominator_	*Ruminococcaceae*	21
		*Lachnospiraceae*	14
		*Clostridiales* vadin BB60 group	6
		*Lactobacillaceae*	6
		*Christensenellaceae/Eggerthellaceae/*	
		*Erysipelotrichaceae/Bacillaceae/*	
		*Enterococcaceae/Clostridiales* Family XIII/	
		*Defluviitaleaceae/Clostridiales*	1
Cobb	y14_denominator_	*Ruminococcaceae*	39
		*Lachnospiraceae*	17
		*Clostridiales* vadin BB60 group	14
		*Mollicutes* RF39	4
		*Christensenellaceae*	4
		*Peptococcaceae/Clostridiales* Family XIII	2
		*Bacillaceae/Erysipelotrichaceae/*	
		*Eggerthellaceae/Clostridiaceae/* 1	
		*Defluviitaleaceae/Coriobacteriaceae/*	
		*Clostridiales*	1

For the comparison between Cobb and Ross, the covariate Breed accounted for 15.3% of variance. Log ratio balances y2 (β = −13.7, *p* = 0.01) and y14 (β = 12.2, *p* = 0.001) were significant predictors for the covariate of breed. The log ratio of balance y2 was, on average, lower in Ross showing a higher relative abundance of denominator ASVs. This difference in abundance is visible on the dendrogram heatmap (Supplementary Figure 3). The log ratio of balance y14 was lower in Cobb. The dendrogram heatmap shows that this is likely due to an increased relative abundance of y14_denominator_ ASVs in Cobb samples. ASV taxonomy of these balances is displayed in Table 5.

For the comparison between Hubbard and Ross, the covariate breed accounted for 12.4% of variance. Log ratio balance y1 was approaching significance (β = 8.22, *p* = 0.07) and on review of the dendrogram heatmap (Supplementary Figure 4) was included in the analysis. The log ratio of balance y1 was lower in Hubbard samples. This difference was due to an increased relative abundance of y1_denominator_ ASVs which can be seen in the dendrogram heatmap. These ASVs were mainly assigned to *Ruminococcaceae* (*n* = 21), *Lachnospiraceae* (*n* = 7) and *Clostridiales* vadin BB60 group (*n* = 6). The remaining ASVs were assigned to *Christensenellaceae* (*n* = 2) and one each to *Peptococcaceae, Enterococcaceae, Clostridiales* Family XIII and *Eggerthellaceae*.

For the comparison between Hubbard and Cobb, the covariate breed accounted for 9.7% of variance. Log ratio balance y15 (β = 4.13, *p* = 0.01) was a significant predictor for the covariate of breed. The log ratio of balance y15 was lower in Cobb suggesting that y15_denominator_ ASVs are more abundant. This is confirmed by the dendrogram heatmap (Supplementary Figure 5). The ASVs composing y15_denominator_ were assigned to *Ruminococcaceae* (*n* = 3) and *Coriobacteriaceae* (*n* = 1).

## 4. Discussion

### 4.1. Bacterial Colonization of the Caecum

This study aimed to characterize the succession of bacteria in the caecal microbiota in three broiler breeds between 0 and 42 days post hatch. Although the results of this study must be interpreted in light of small sample sizes which limit statistical power, and the pooling of samples, which obscures the inherent variability of individual microbiota composition, broad patterns are visible within the data and merit further discussion. The initial microbiota observed in Hubbard and Ross breeds was very similar to that described in other studies (8). Large differences in microbiota composition were observed between breeds immediately post hatch. This is most likely due to different bacterial exposure during handling of the chicks from hatch to collection (7). These early differences in microbiota composition did not affect the subsequent development of a mature microbiota. Differences between breeds were still visible at 3 and 7 d.p.h but were no longer visible at 14 d.p.h. This suggests that environmental exposure, diet and management practices are more important than genetics in shaping the caecal microbiota.

The rise of *Bifidobacteriaceae* at 3.d.p.h may represent an important step in the maturation of the caecal microbiota as a stimulus for *Bacteroidaceae* growth. Recent studies have shown that *Bacteroides fragilis* and other intestinal bacteria can metabolize exopolysaccharides, a complex carbohydrate produced by some *Bifidobacterium* strains (36, 37). It's possible that an initial rise in *Bifidobacterium* produces polysaccharides which are then used by other bacteria as a substrate and the basis for the expansion of their populations. Salazar et al. (37) also showed that exopolysaccharides from different strains of *Bifidobacterium* supported populations of different bacteria such as *Faecalibacterium prausnitzii*. This suggests that the initial colonizing strains of *Bifidobacteriaceae* could influence future microbiota development in the caecum.

As well as promoting the growth of other caecal microbes *Bifidobacteriaceae* play an important role in pathogen exclusion and intestinal barrier function (38, 39). Most of the evidence for this comes from mammalian studies, however, a *Bifidobacterium* probiotic has been shown to improve epithelial integrity in chickens (40). The mechanism may be associated with *Bifidobacterium*s production of acetate which can protect mice epithelial cells in the face of *E. coli* O157 infection (41). *Bifidobacterium* also plays a role in dendritic cell maturation and the balancing of regulatory T cell and T helper 17 cell development (42, 43), similar to the role of *Candidatus* Savagella in the ileum.

One of the caecums main functions is bacterial fermentation of indigestible polysaccharides to produce short chain fatty acids which can be utilized by the host's epithelial cells. The taxa which fulfil this role are certain classes of *Firmicutes* (such as *Lachnospiraceae* or *Ruminococcaceae*) and *Bacteroidetes*. In this study, they were equally abundant with some *Firmicutes* localizing to mucus and *Bacteroides* to the lumen. Previous studies have found that one or the other is more abundant in the caecum (12–14). This discrepancy between studies may be explained by differences in methodology or sampling. However, it has been reported that even among chickens from the same flock, the *Firmicutes*:*Bacteroidetes* (F/B) ratio can vary substantially (44). There is conflicting evidence as to the F/B ratio's impact on metabolism and feed conversion ratio (FCR). Stanley et al. (44) reported that the differences found between chickens in the same flock didn't appear to have an effect on apparent metabolizable energy or FCR. In contrast to this result, a comparison of the faecal microbiota between high and low FCR broiler chickens at 49 d.p.h found significant differences in microbiota composition between the two groups. Low FCR birds had a higher F/B ratio than high FCR birds suggesting that a higher relative abundance of *Firmicutes* can be associated with increased metabolic efficiency (45). Similar patterns have been noted in the human microbiota with a higher F/B ratio linked to obesity (46). However, *Firmicutes* is a diverse phylum and closer inspection at a lower taxonomic level reveals a more complex pattern. The two major families of caecal *Firmicutes* have been identified as *Ruminococcaceae* and *Lachnospiraceae*. Singh et al. (45) found that the relative abundance of *Lachnospiraceae* was nearly two times higher in high FCR birds while the relative abundance of *Ruminococcaceae* was 15 times higher in low FCR birds.

*Lachnospiraceae* was the first *Clostridia* to colonize the caecum in this study. In contrast, *Ruminococcaceae* was a relatively late colonizer which appeared to replace *Lachnospiraceae*, especially in the lumen, at later time points. These two families are poorly classified, and material related to their potential role in the microbiota is scarce. Differences in gene abundance between genomes of *Lachnospiraceae* and *Ruminococcaceae* have been noted (47). However, the functional significance of these differences in relation to the pattern of succession is not clear. One exception to this rule is *Faecalibacterium prausnitzii*, a butyrate producing member of *Ruminococcaceae* (48, 49), which has been identified as a potentially beneficial microbe (50). Aside from its prominence as a late stage member of the caecal microbiota little is known about the impact of *F. prausnitzii* on the chicken. Some inferences can be made from observations reported in mouse and human studies. In these species, *F. prausnitzii* is thought to have an anti-inflammatory effect since lower counts are observed in various inflammatory diseases (51, 52). Additionally, inflammation and intestinal barrier function are improved in a mouse IBD model by the addition of *F. prausnitzii* (51, 53). While the production of butyrate may play a role in *F. prausnitzii*s beneficial effects, the possibility of a more complex mechanism involving the production of other molecules should not be ruled out (54). The results also showed later colonization of the caecum by other members of *Firmicutes* such as *Christensenellaceae, Clostridiales* vadin BB60 group, *Peptococcaceae* and Bacillaceae, as well as *Mollicutes* RF39. Some, for example *Clostridiales* vadin BB60 group, are poorly classified and little is known about their metabolism or role in the microbiota. One exception is the family *Christensenellaceae* which has been linked to lower BMI and overall gut health in humans (55, 56).

Another feature of the developing microbiota in this study was the decrease in *Enterobacteriaceae* over time. This bacterial family is considered of importance in poultry production, not just as pathogens, but also due to their carriage of genetic factors responsible for antimicrobial resistance such as extended-spectrum beta-lactamase genes (57). The relative abundance of *Enterobacteriaceae* was highest at 0 and 3 d.p.h before decreasing to its lowest relative abundance at 21 d.p.h. A similar pattern of replacement of *Enterobacteriaceae* by other taxa over the few weeks of life has been observed in previous studies (8, 15, 58). Reduced abundance and growth of *Enterobacteriaceae* have been linked to levels of SCFAs like acetate, butyrate and propionate both *in vitro* and *in vivo* (59). As such, the reduction in *Enterobacteriaceae* observed in this study and others could be attributed to caecal colonization by *Lachnospiraceae, Ruminococcaceae* and *Bacteroidaceae*. This highlights the importance of these taxa in areas other than metabolism and suggests that interventions which promote early colonization by these taxa should be prioritized.

The order of caecal bacterial succession described in this paper may not be representative of a commercial setting where environmental exposure and other factors will allow for a differing microbiota to develop. However, it does raise questions about the general mechanisms of succession. It's possible that the late arrival of taxa such as *Clostridiales* vadin BB60 group and *Bacillaceae* was a question of environmental exposure, that these taxonomic groups were not present in the environment until 14 or 21 d.p.h. However, it also raises the possibility that these taxa have some prerequisite conditions that must be fulfilled, either by the host or earlier bacterial colonizers, before they can establish a population in the caecum. Identifying factors or interventions that accelerate the development of a mature microbiota may provide further benefits to chicken production in terms of increased yields or reduced losses to infectious disease.

### 4.2. The Mucus and Lumen-Associated Microbiota Are Different

Similar differences between mucus and lumen microbiota have been described in other species. Higher levels of *Lachnospiraceae* and a lower abundance of *Bacteroidaceae* have been reported in mouse colon mucus when compared to the lumen (32). This may be due to the differing energy sources of these bacteria. As in other animals, bacterial degradation of indigestible polysaccharides into short chain fatty acids (SCFA) plays an important role in host nutrition. In the human gut, *Bacteroides* have the highest number and diversity of genes associated with polysaccharide metabolism (60). This is also true of chickens with metagenomics studies finding polysaccharide utilization systems associated with caecal *Bacteroidetes* (1). It would make sense for *Bacteroides* abundance to be higher in the lumen where their energy source is most abundant. The same reasoning can explain the higher abundance of *Lachnospiraceae* in mucus. A recent metagenomic analysis of *Lachnospiraceae* and *Ruminococcaceae* genomes showed differences in gene abundance related to carbohydrate metabolism. *Ruminococcaceae* have higher numbers of cellulase and xylanase genes related to fermentation of substrates which are more abundant in the lumen. *Lachnospiraceae* were found to have a higher number of genes for glycoside hydrolase 13, an enzyme associated with cleavage of α-amylase bonds present in starch and glycogen (47).

Members of the *Lachnospiraceae* family have also been shown to utilize mucin glycans as a sole carbon source, producing propanol and propionate (61). Due to the preference for chicken *Lachnospiraceae* to reside in the mucus, it is likely that these strains also possess enzymes which allow them to utilize host mucins as an energy source.

Equally, strains of *Coriobacteriaceae* and *Bifidobacteriaceae* have also been found with the ability to degrade mucin in other species (62, 63). The ability of *Bifidobacteriaceae* to adhere to mucus is well documented and also explains higher abundance in the mucus than the lumen (64–66)

The host likely benefits from abundant *Firmicutes* in mucus. Differences in SCFA production have been observed between *Firmicutes* and *Bacteroides* in the chicken caecum with the former producing butyrate while the latter produces mainly propionate (67). Since, epithelial cells utilize butyrate as their principal energy source, a ready source in the mucus is likely to support early epithelial maturation and growth (67).

While these results can be explained by bacterial adaptation to niches based on substrate availability, an alternative explanation may lie in host regulation of the microbiota and maturation of the immune system. In this study, there was a pattern of mucosal colonization by ASVs which appear in the caecum before 14 d.p.h. While the association between appearance in the microbiota and the ability to colonize caecal mucus may be incidental since *Lachnospiraceae* colonize the caecum before *Ruminococcaceae*, it may be that early presence in the caecum results in host tolerance of certain ASVs allowing them to occupy the mucus layer.

These results have implications for other studies of the chicken caecal microbiota. For example, studies observing the effects of probiotics containing *Bifidobacteriaceae* or *Bacillus* which observe only the luminal microbiota may miss increased abundance of these taxa. Additionally, the *Firmicutes*:*Bacteroides* ratio is often used to compare microbiota between groups (46, 68, 69). One notable study using this parameter found a large range in *Firmicutes*:*Bacteroides* ratio among individuals of the same breed housed under the same conditions (44). It is possible that some of this variation could be due to different ratios of lumen content to mucus in samples. This would be particularly noticeable in chickens which had empty caeca at the time of sampling since any content present is likely to have a high proportion of mucus. Many studies of the caecal microbiota do not detail the sampling methodology enough to discern whether samples were taken from the lumen or the mucus. These results further emphasize the importance of a detailed methodology which discerns between sampling from different compartments of the caecum.

### 4.3. Differences Between Breeds

Some differences between breeds were visible in the pooled samples. These differences occurred at 0 and 3 d.p.h with the greatest difference at 0 d.p.h. At subsequent time points there were no significant differences between pooled samples from different breeds. The difference in early microbiota can be explained by the previous finding that chicks from different hatcheries are colonized by different microbes (7). Although the chicks used in this experiment were sourced from a single hatchery, chicks from different breeds are hatched and reared in different buildings resulting in different environmental exposure. As such, the differences between breeds at 0 d.p.h cannot be attributed to genotype since it is likely that chicks are colonized by whichever environmental bacteria are present at the time of hatch and this will vary between hatcheries and within hatcheries.

Analysis of five samples of caecal mucus taken from each breed at 42 d.p.h showed differences in microbiota between breeds. The results of alpha and beta diversity, as well as Gneiss analysis, suggest that Cobb and Hubbard had the most similar microbiota composition with the greatest differences found between Cobb and Ross. Many of these ASVs that were differentially abundant between Cobb and Ross were assigned to *Ruminococcaceae* and *Lachnospiraceae*, perhaps as these were among the most prevalent taxa in the caecum. It is of interest that most *Lactobacillaceae* were more abundant in Ross samples while a high proportion of *Christensenellaceae* were more abundant in Cobb. Both of these taxa have been linked to metabolic effects on the host. Dietary supplementation with *Lactobacillus* strains has been previously demonstrated to improve body weight gain and FCR in chickens (70) while *Christensenellaceae* is more abundant in the microbiota of lean compared to obese humans (55, 56). However, in the absence of detailed performance data such as FCR or body composition analysis, it is not possible to ascribe a functional or practical significance to these results. Greater taxonomic resolution or metagenomic analysis would also be required as the metabolic effects of bacteria is likely to be strain dependent.

Another factor which may have affected these results is the relative composition of lumen contents to mucus of the samples. It was noted earlier that the pooled lumen sample from Cobb at 42 d.p.h was the only lumen sample with a similar beta diversity to mucus samples. Since it has already been demonstrated that there are significant differences between lumen and mucus microbiota, the observed difference could be attributed to a random sampling error amplified by the small sample sizes used in this study. It is possible that samples taken from Ross had more content or less mucus compared to those from Cobb, accounting for the differences between them. Caecal emptying occurs several times a day in chickens. A recently emptied caecum would have a higher proportion of mucus to luminal contents when expressed during sampling which may yield different results had the caecum been full. With this in mind, while this experiment showed some differences in microbiota composition between genotypes, clear results are hampered by small sample size. However, the influence of genotype on caecal microbiota cannot be discounted.

## 5. Conclusion

This study observed the development of the caecal microbiota between hatch and 42 d.p.h, covering the lifespan of a modern broiler chicken. After hatch, the microbiota had poor diversity and was mainly composed of environmental bacteria. Between hatch and 21 d.p.h, the microbial community became more complex and matured to a stable, diverse microbiota. It is worth noting that for more than half of the production period the microbiota was developing and would be more susceptible to changes brought about by external factors. Earlier interventions in the microbiota are likely to be more successful, both in terms of altering the mature microbiota and optimizing the microbiota's impact on immune system maturation and metabolic function. Interventions should focus on promoting early maturation particularly with respect to *Bacteroidaceae, Lachnospiraceae* and *Ruminococcaceae*. Although some significant differences were found in the microbiota between breeds, it is possible that these differences were caused by other factors such as a higher relative composition of luminal content to caecal mucus.

## Author Contributions

PR conducted the sampling, sample processing, 16S rRNA gene analysis, interpretation of the results, and wrote the manuscript. MB advised on the design of the experiment and edited the manuscript. JF advised on experimental design and data analysis relating to 16S rRNA gene sequencing. PW advised on experimental design and helped conduct the experiment. All authors read and approved the final manuscript.

### Conflict of Interest Statement

The authors declare that this study received funding from DuPont Industrial Biosciences. The funder advised on the study design and approved the final manuscript for publication but did not have a role in the data collection and analysis or decision to publish. MB was employed by DuPont Industrial Biosciences.

## Supplementary Material

Supplementary Table 1 Ingredient and additive composition of starter and grower diets used in the study.

Supplementary Figure 1 **(A–E)** Taxonomic composition of balances which reveal significant differences between time points.

Supplementary Figure 2 Taxonomic composition of balance y2 which reveal significant differences between mucus and lumen samples.

Supplementary Figure 3 A dendrogram heatmap **(A)** showing differences in the caecal mucus microbiota of Cobb and Ross chickens at 42 d.p.h. The log ratio of balances y2 **(B)** and y14 **(C)** were significantly different between breeds.

Supplementary Figure 4 A dendrogram heatmap **(A)** showing differences in the caecal mucus microbiota of Hubbard and Ross chickens at 42 d.p.h. The log ratio of balance y1 **(B)** was significantly lower in Hubbard chickens due to an increased relative abundance of denominator ASVs.

Supplementary Figure 5 A dendrogram heatmap **(A)** showing differences in the caecal mucus microbiota of Cobb and Hubbard chickens at 42 d.p.h. The log ratio of balance y15 **(B)** was significantly lower in Cobb chickens due to an increased relative abundance of denominator ASVs.
