# Supplementary figures and images for: Development of the Caecal Microbiota in Three Broiler Breeds
Source: Front Vet Sci. 2019 Jun 25;6:201. doi: 10.3389/fvets.2019.00201 (PMC6603203; doi:10.3389/fvets.2019.00201)

**A)**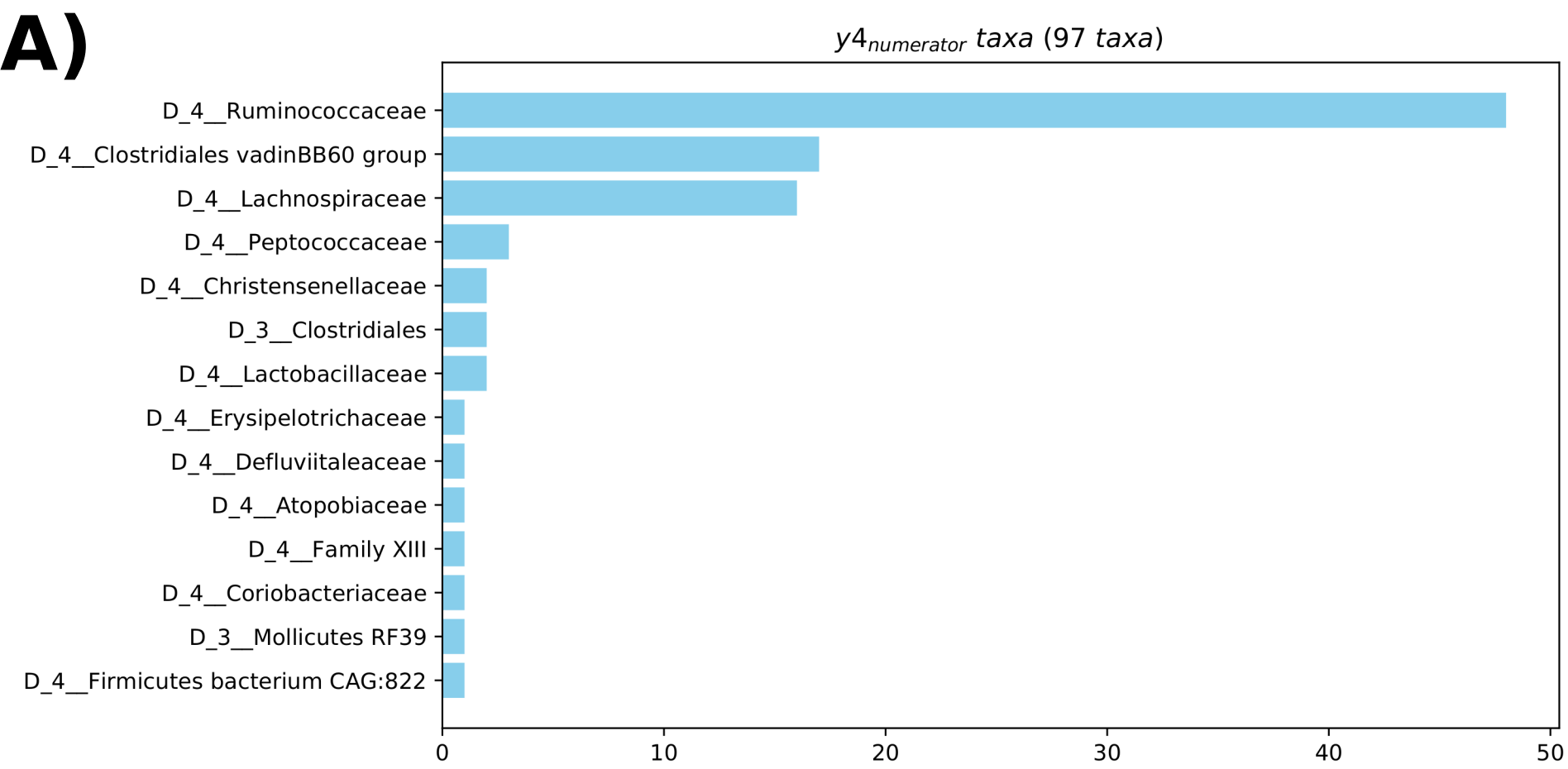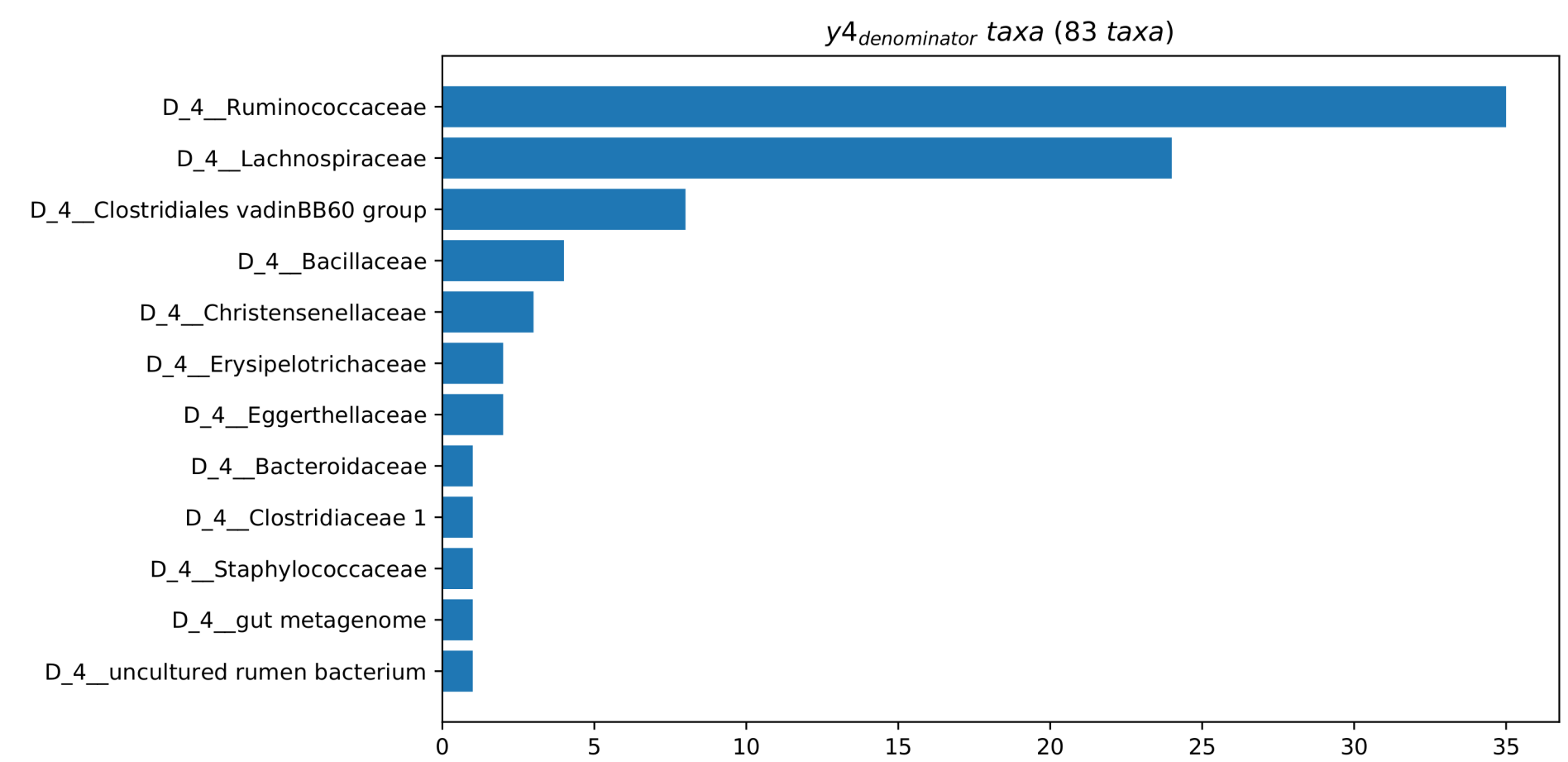**C)**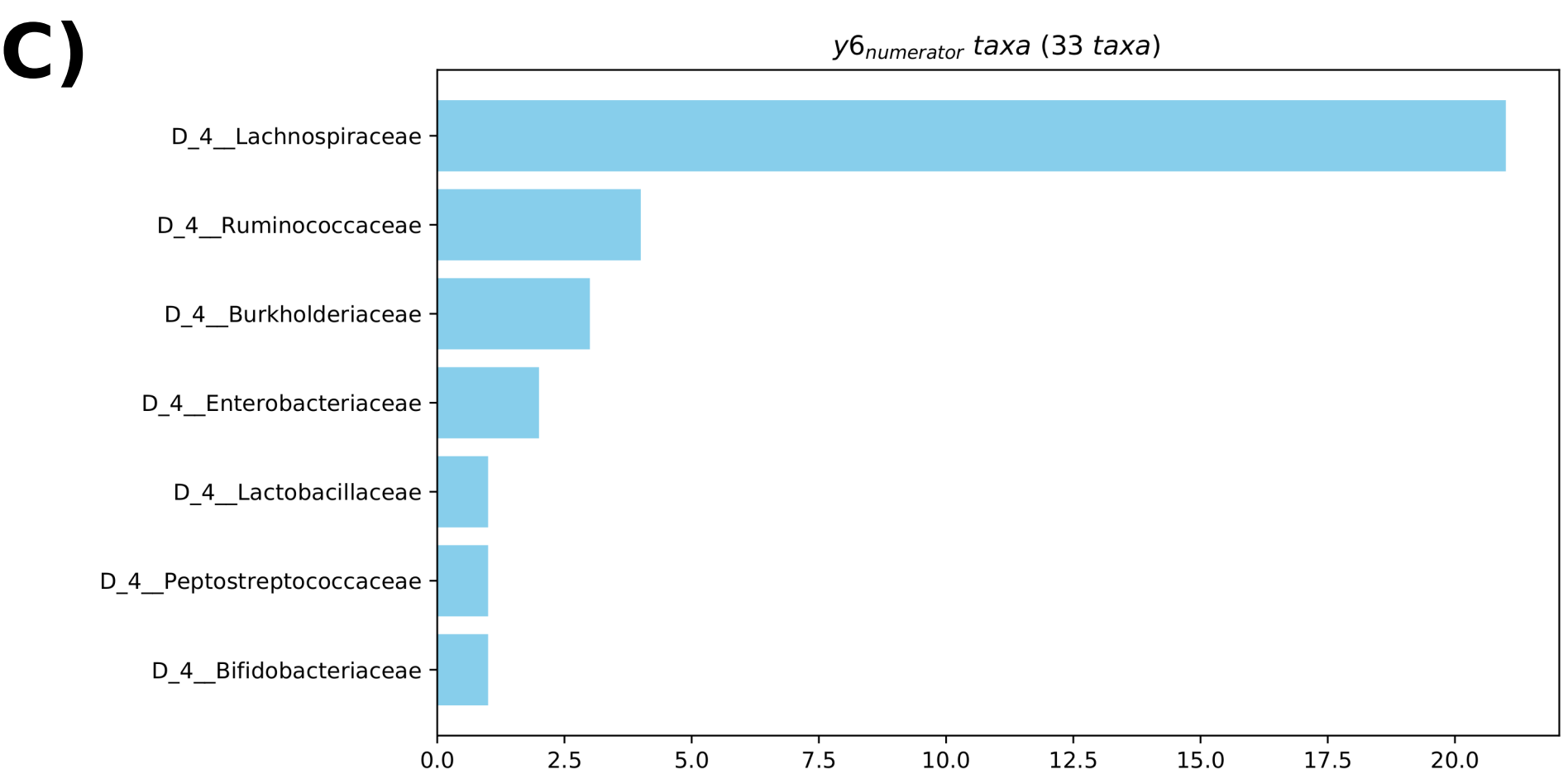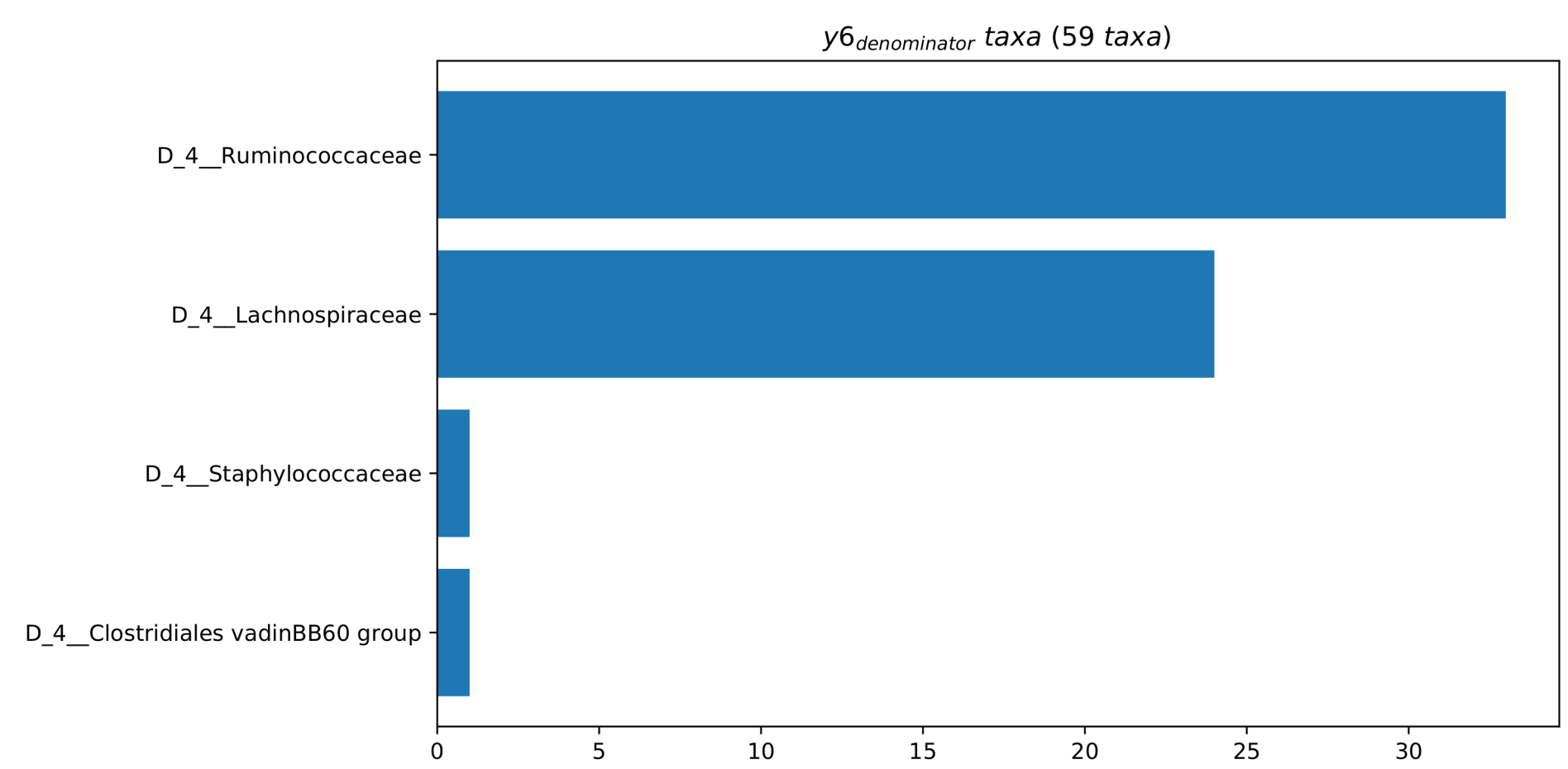**E)**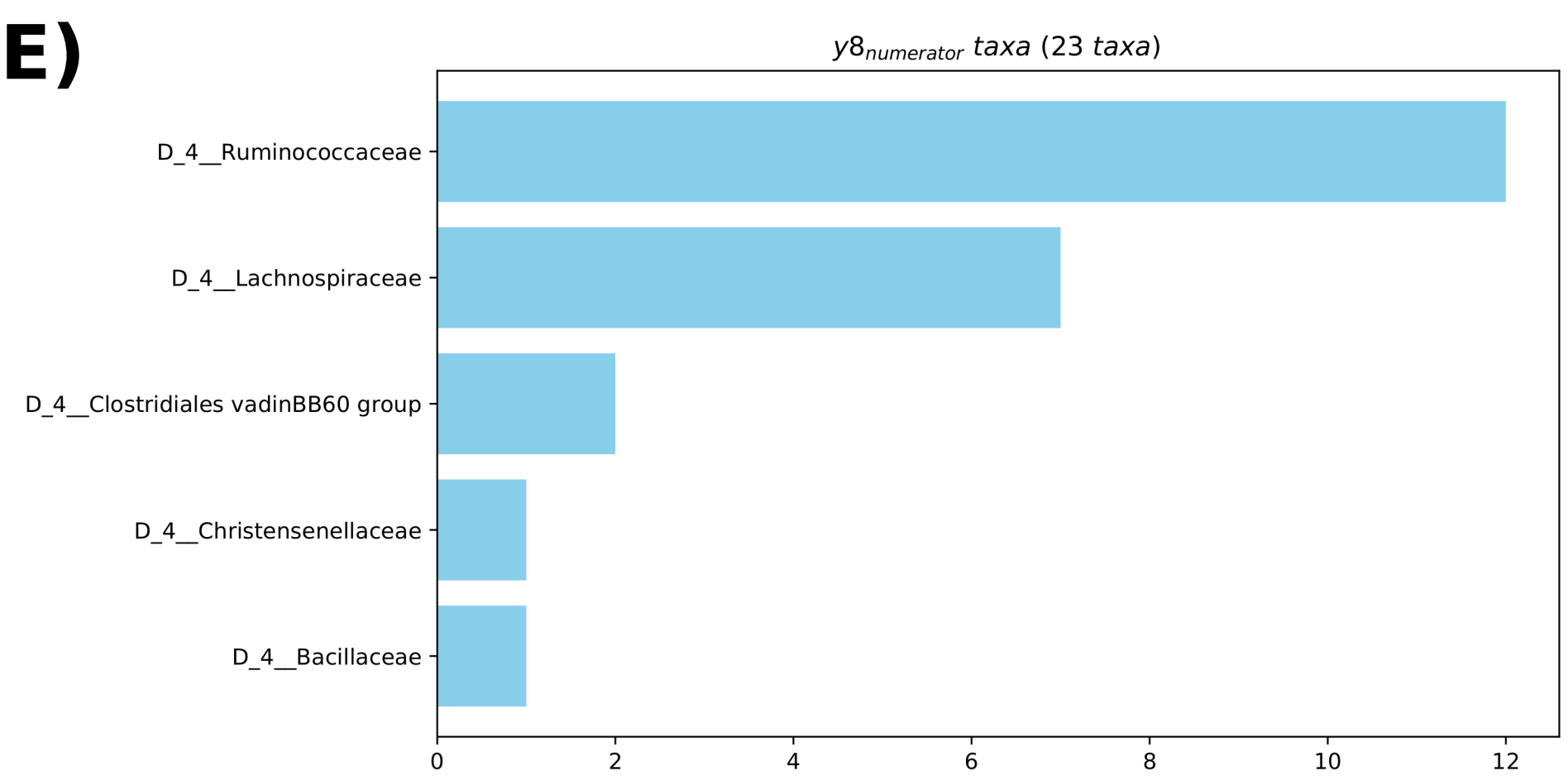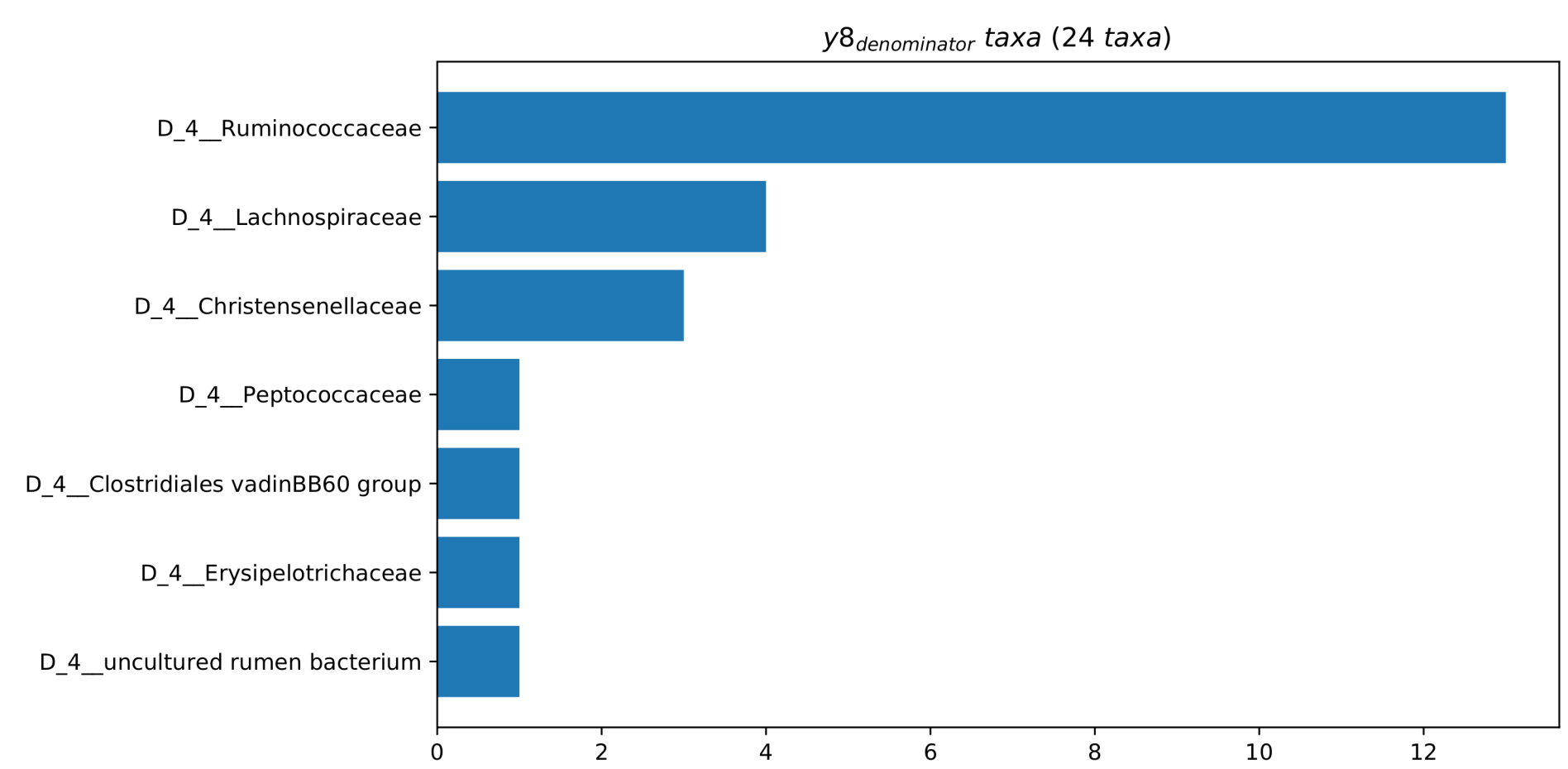**B)**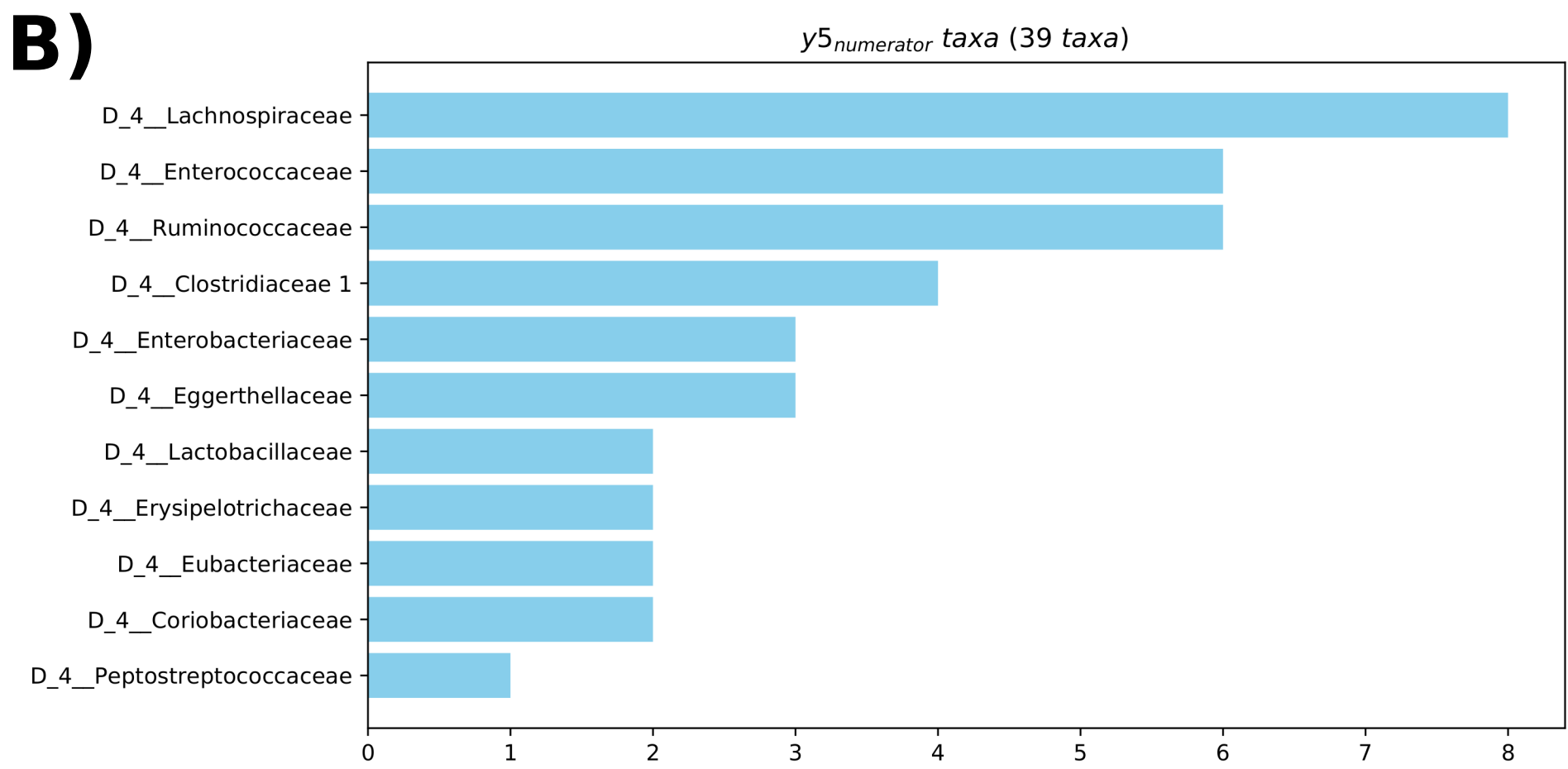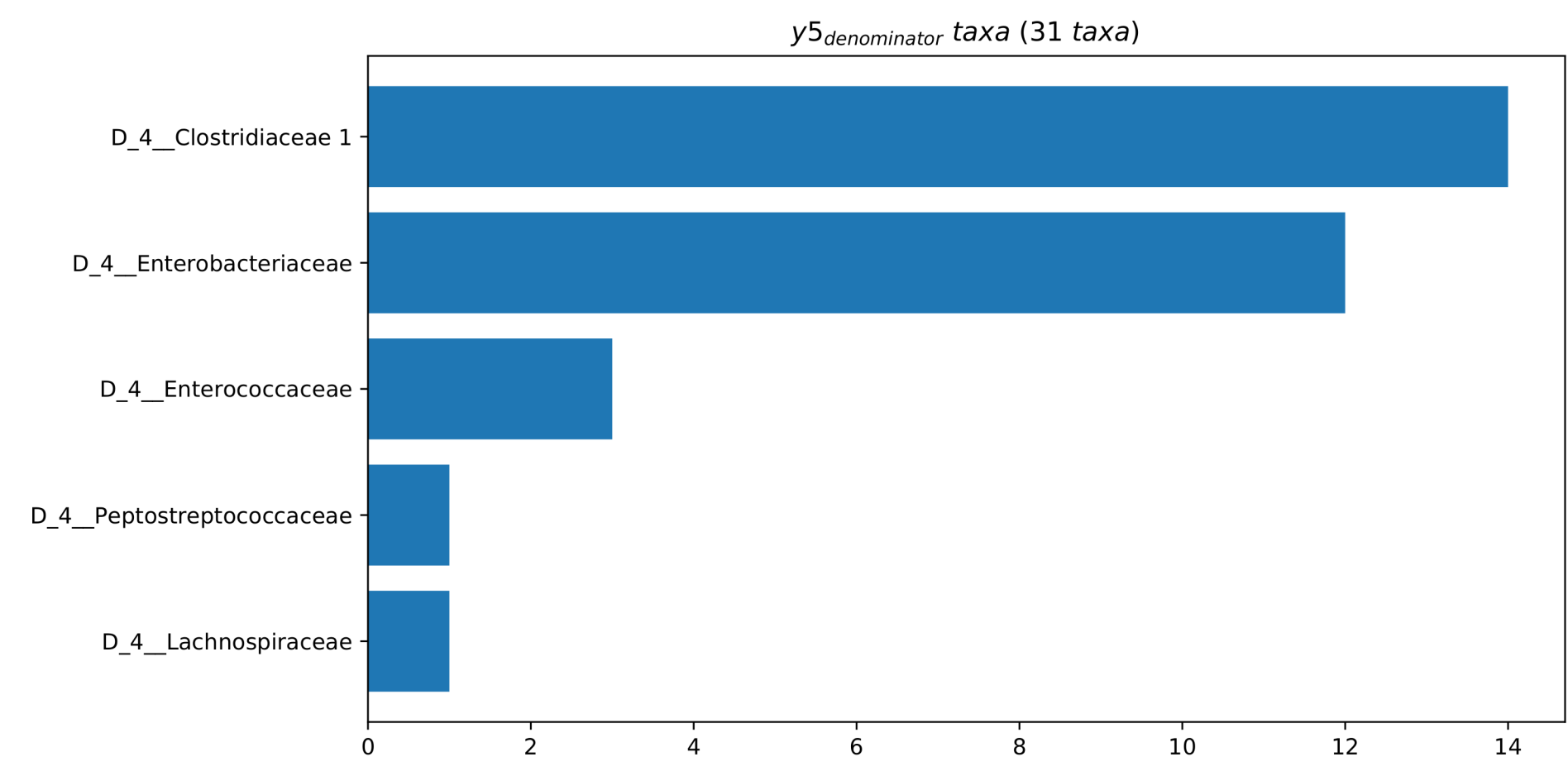**D)**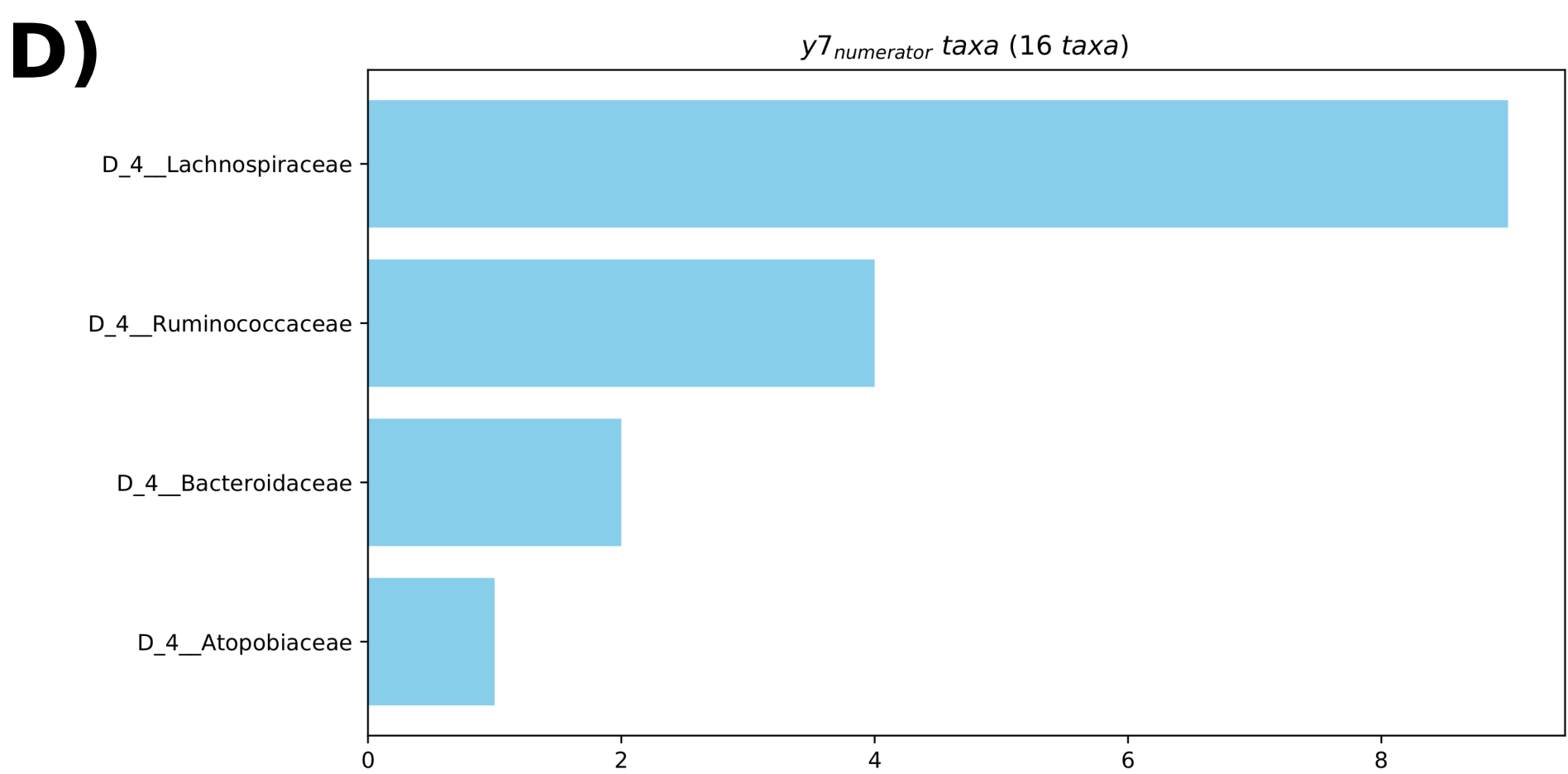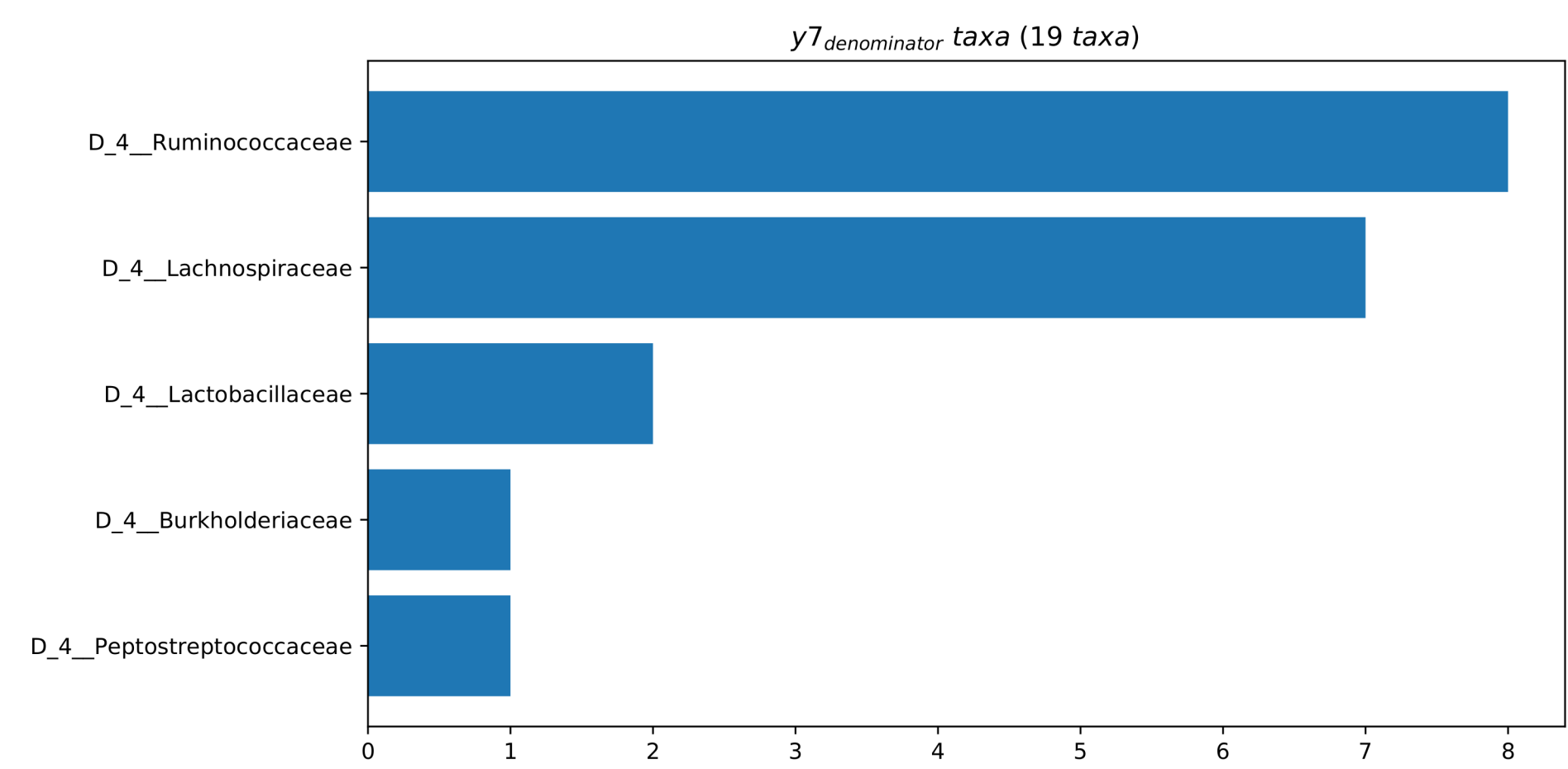

Supplement: Supplementary Figure 1 — (A–E) Taxonomic composition of balances which reveal significant differences between time points. [file Image_1.pdf]

$y2_{numerator}$  taxa (193 taxa)

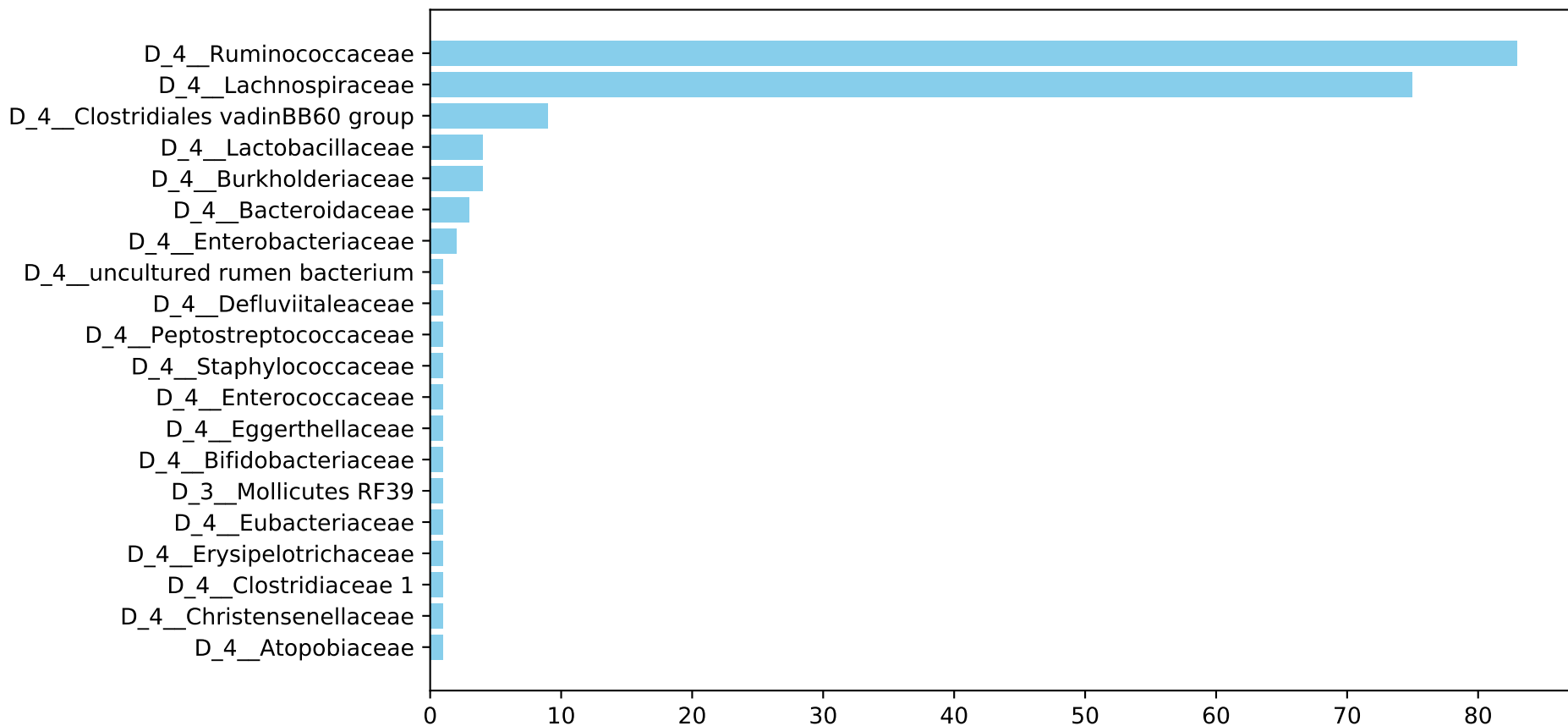

$y2_{denominator}$  taxa (62 taxa)

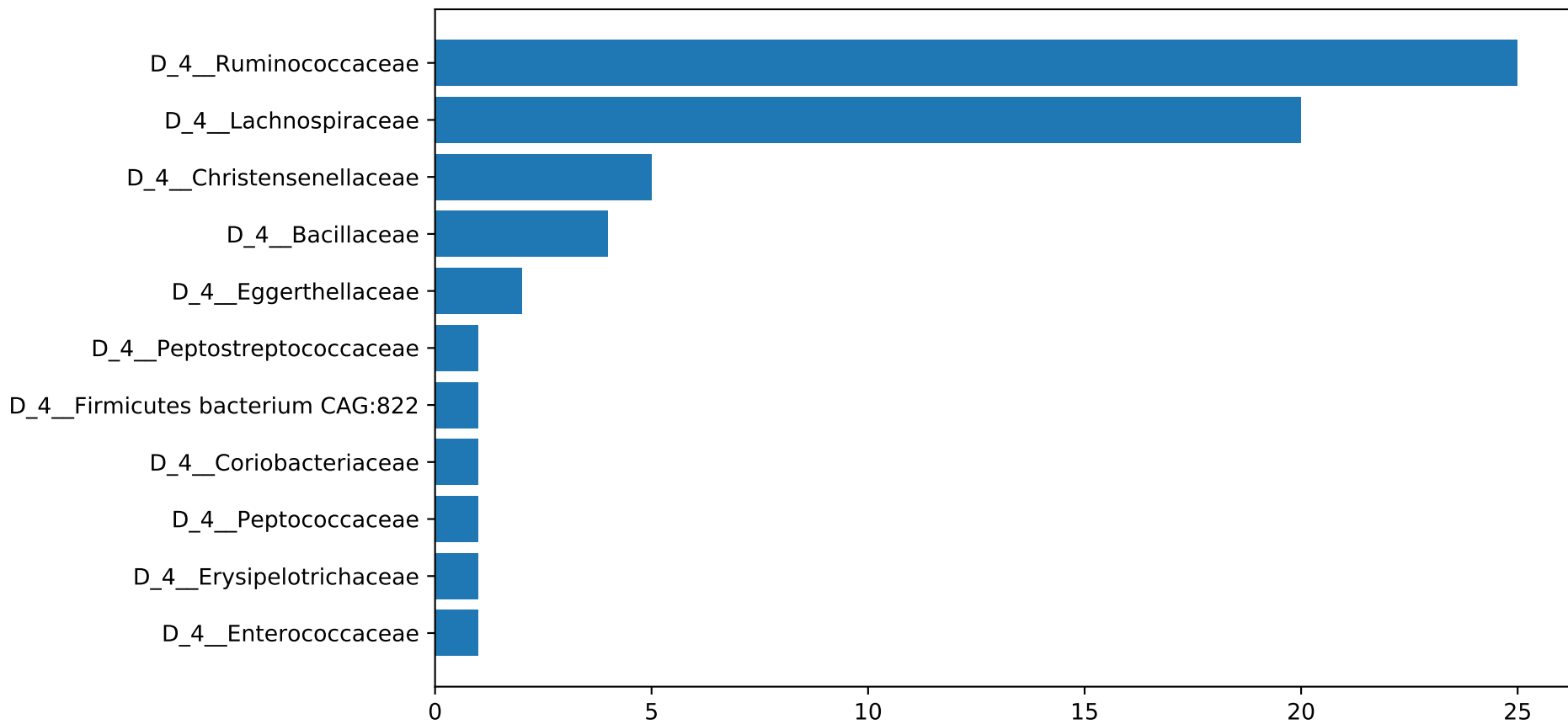

Supplement: Supplementary Figure 2 — Taxonomic composition of balance y2 which reveal significant differences between mucus and lumen samples. [file Image_2.pdf]

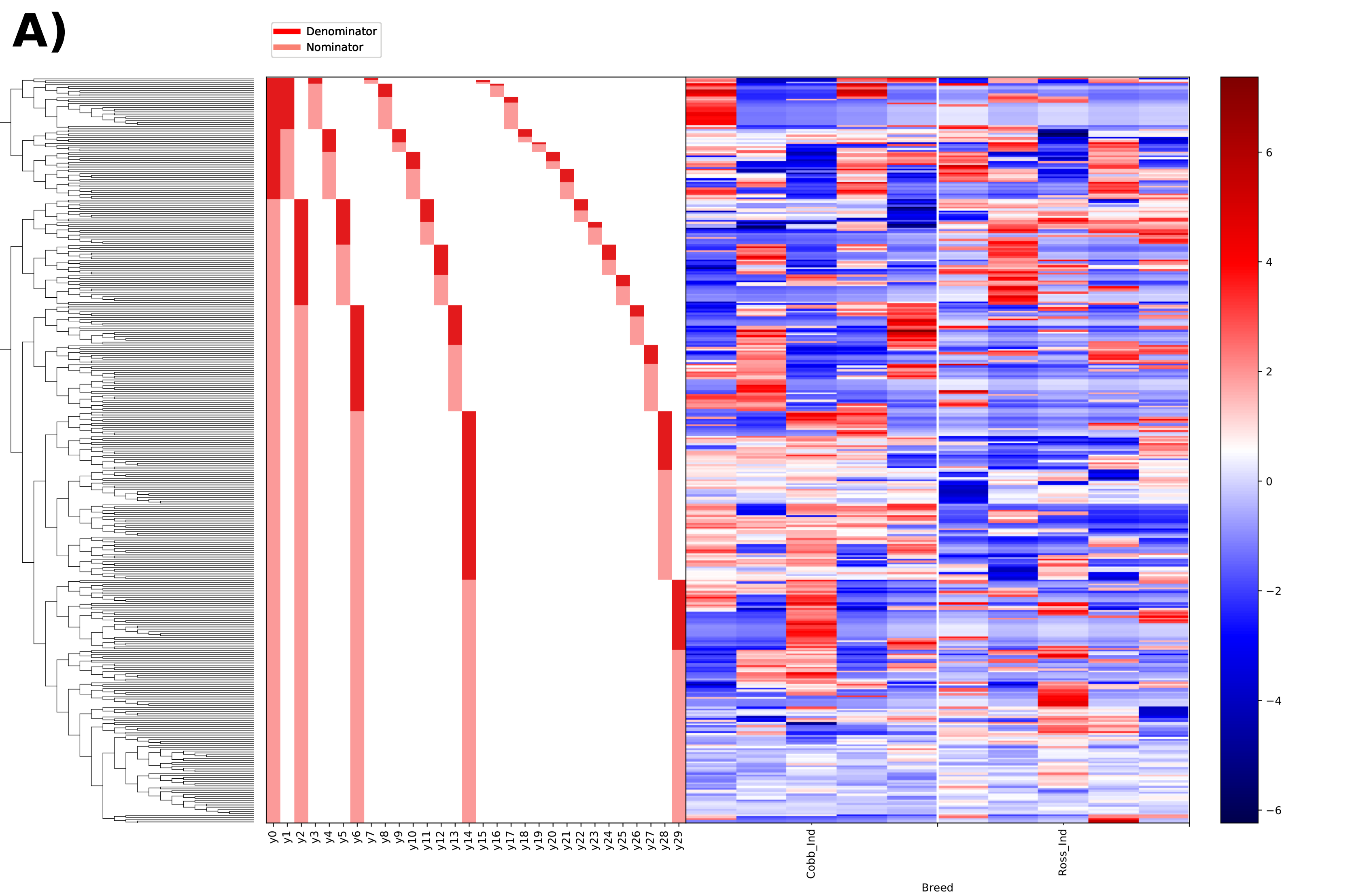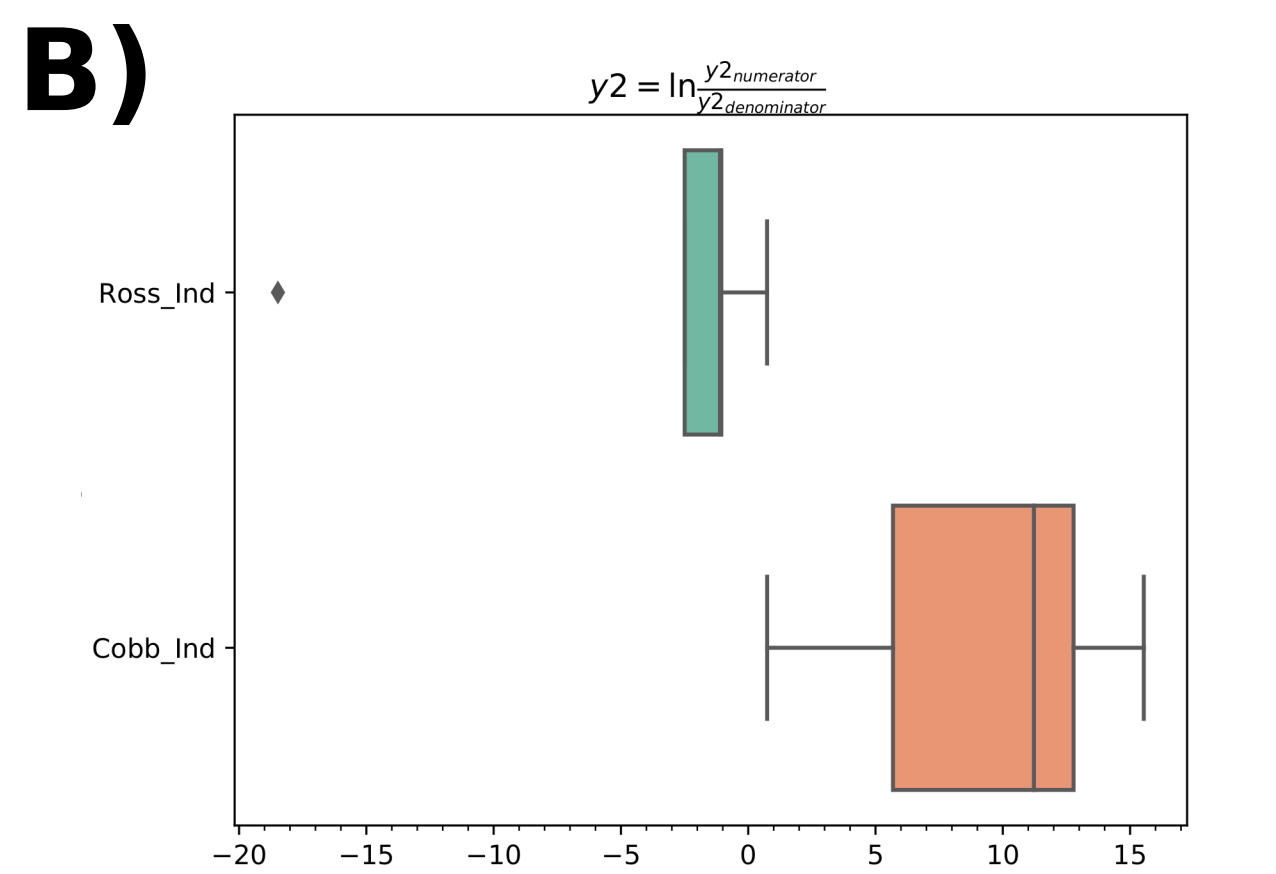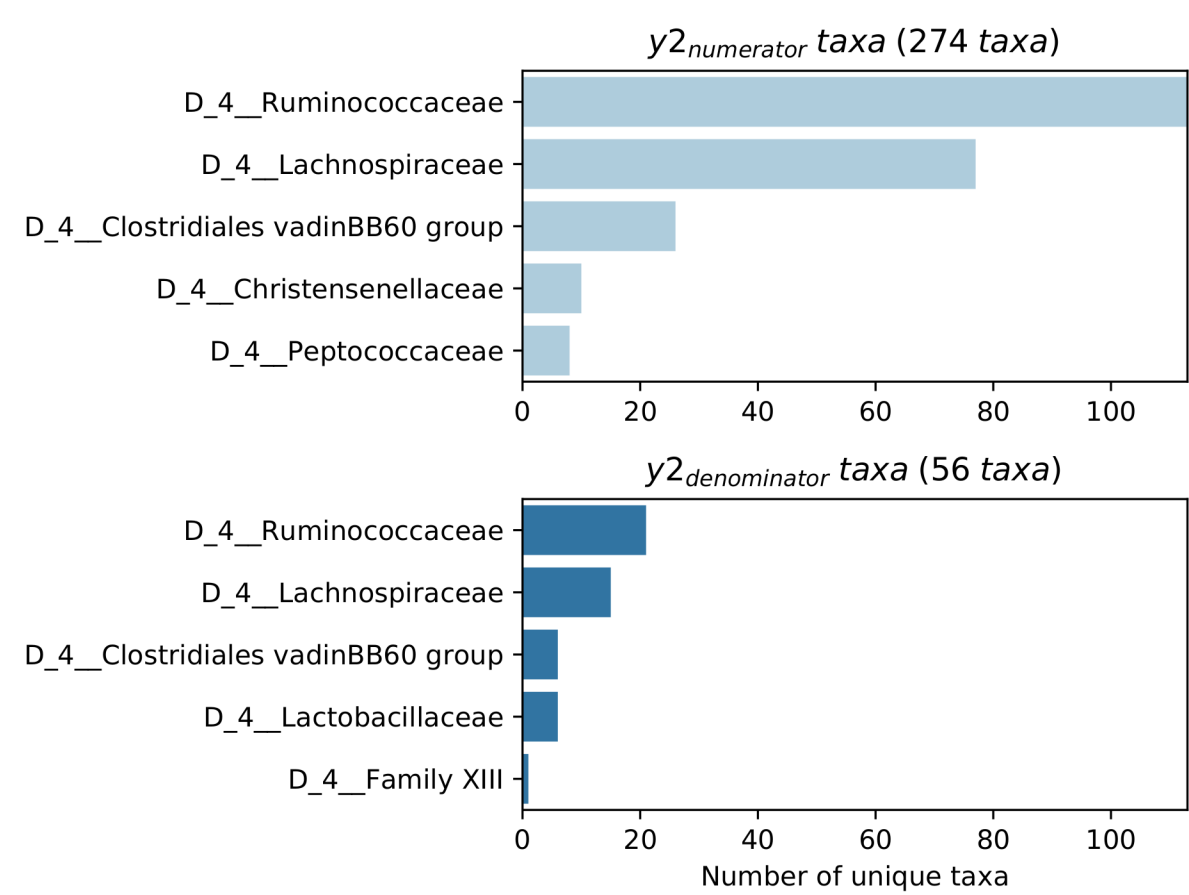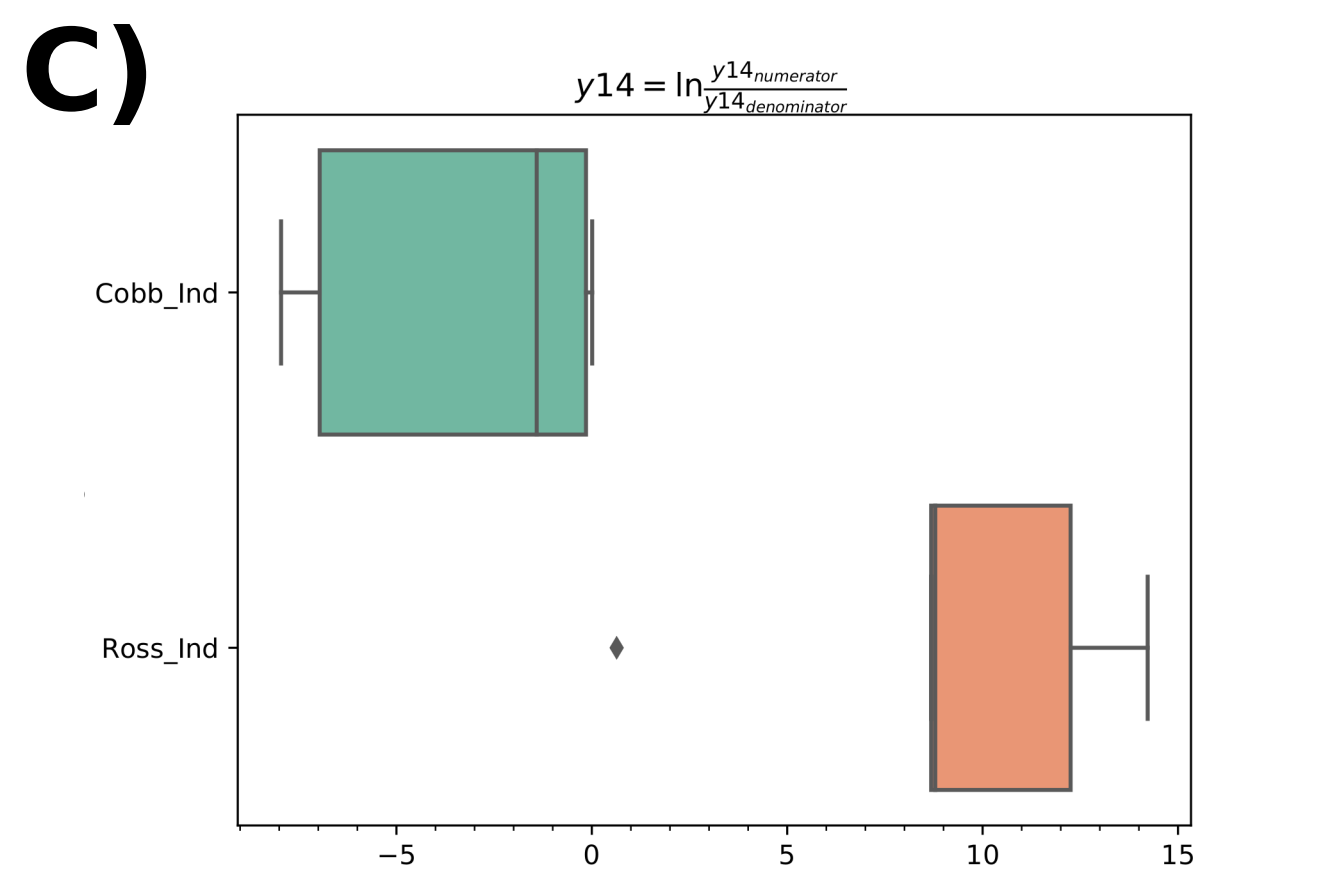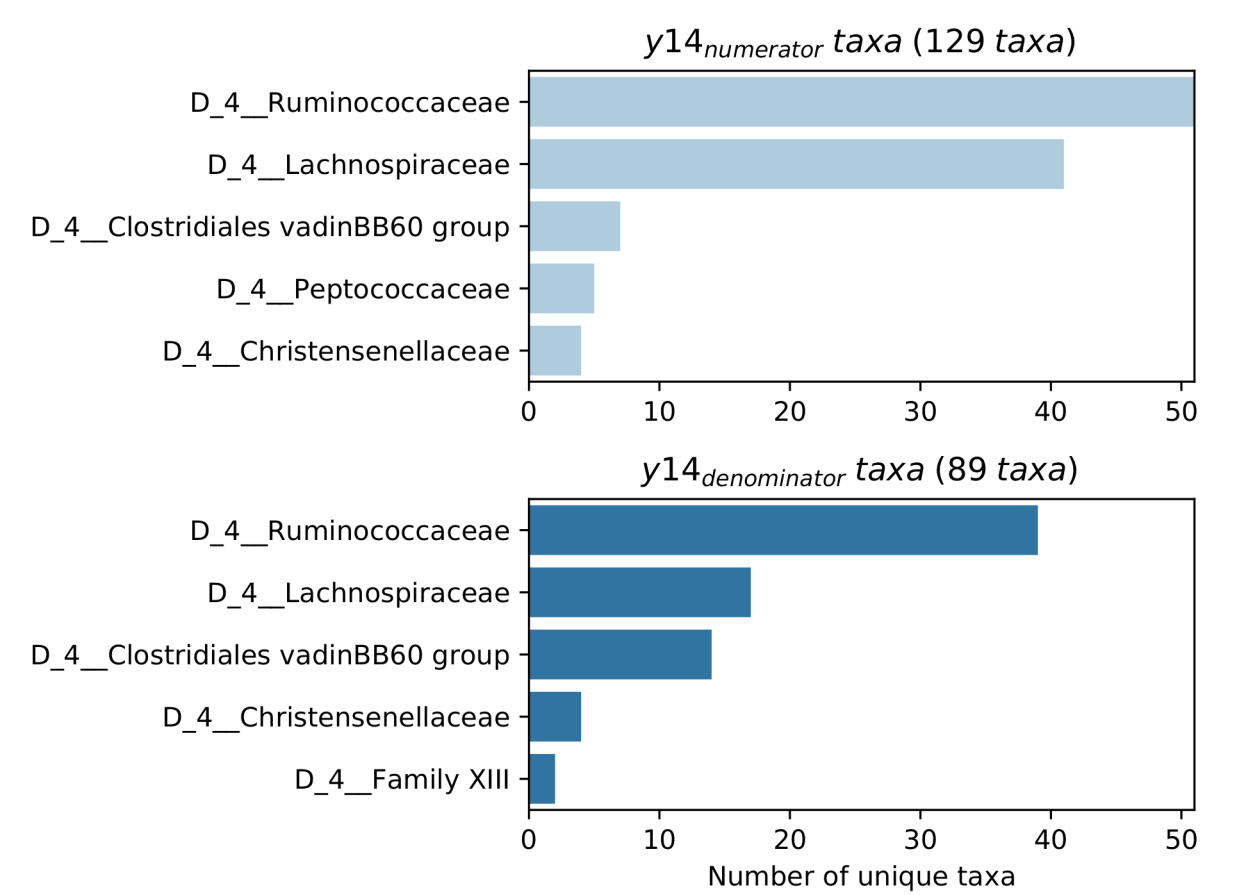

Supplement: Supplementary Figure 3 — A dendrogram heatmap (A) showing differences in the caecal mucus microbiota of Cobb and Ross chickens at 42 d.p.h. The log ratio of balances y2 (B) and y14 (C) were significantly different between breeds. [file Image_3.pdf]

A)

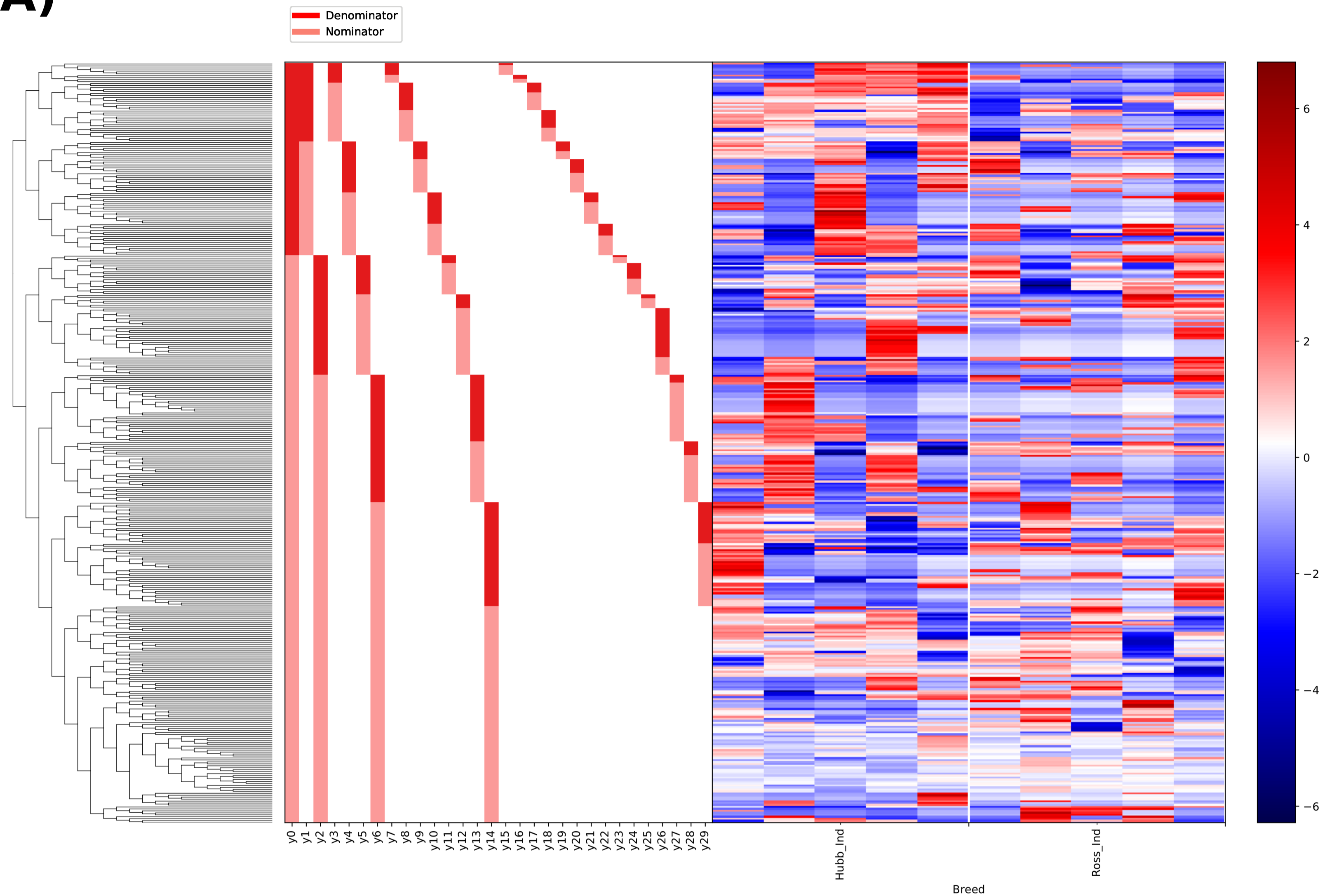

B)

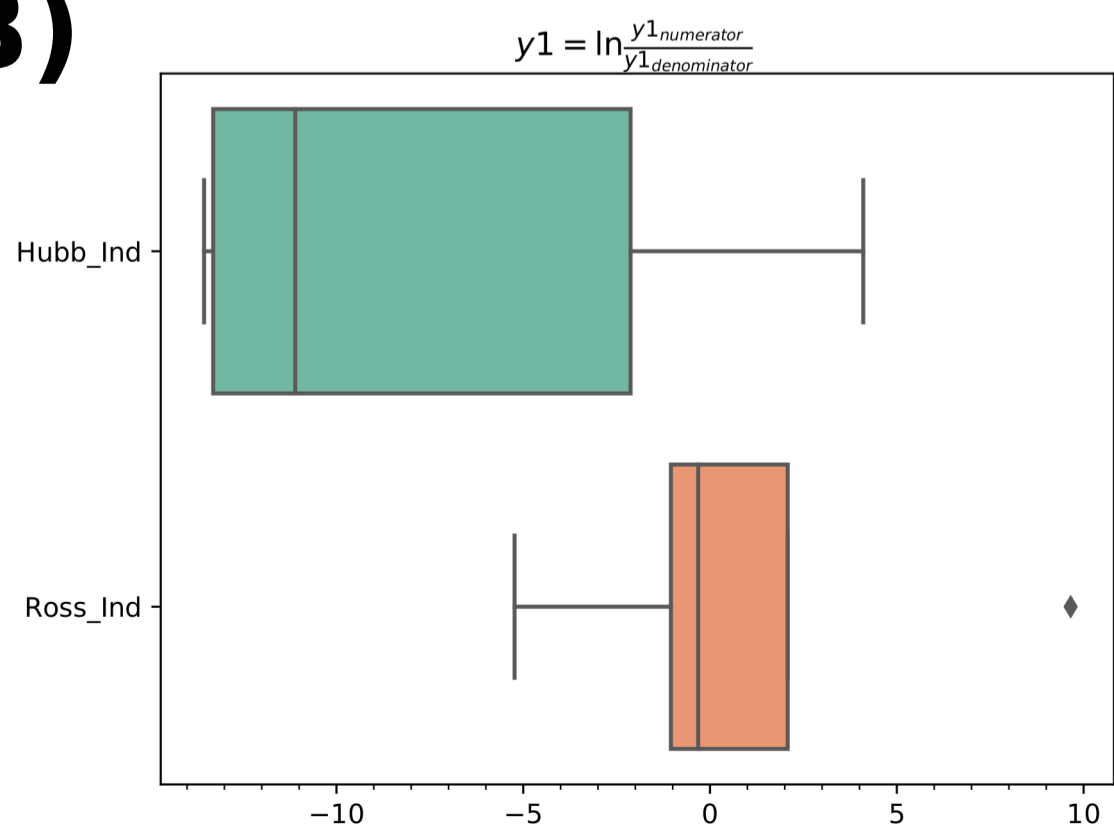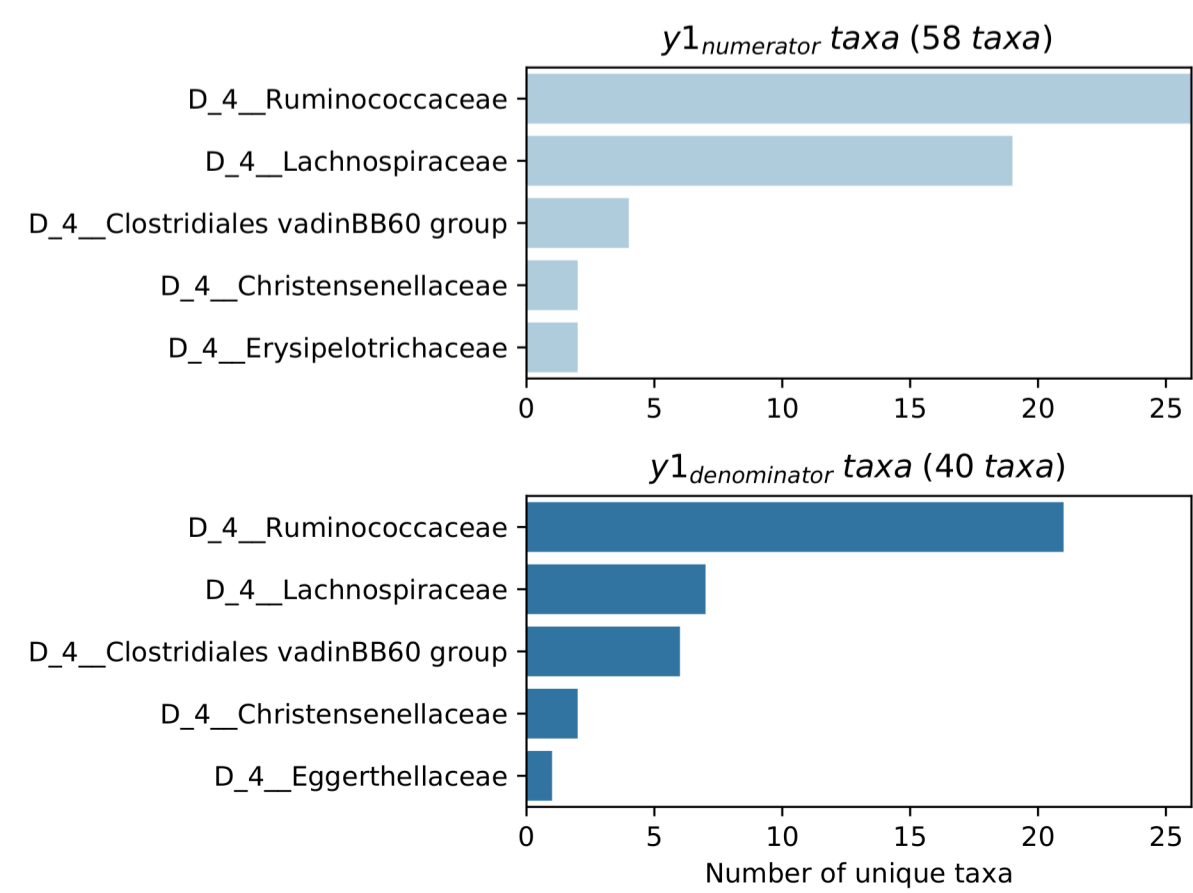

Supplement: Supplementary Figure 4 — A dendrogram heatmap (A) showing differences in the caecal mucus microbiota of Hubbard and Ross chickens at 42 d.p.h. The log ratio of balance y1 (B) was significantly lower in Hubbard chickens due to an increased relative abundance of denominator ASVs. [file Image_4.pdf]

**A)**

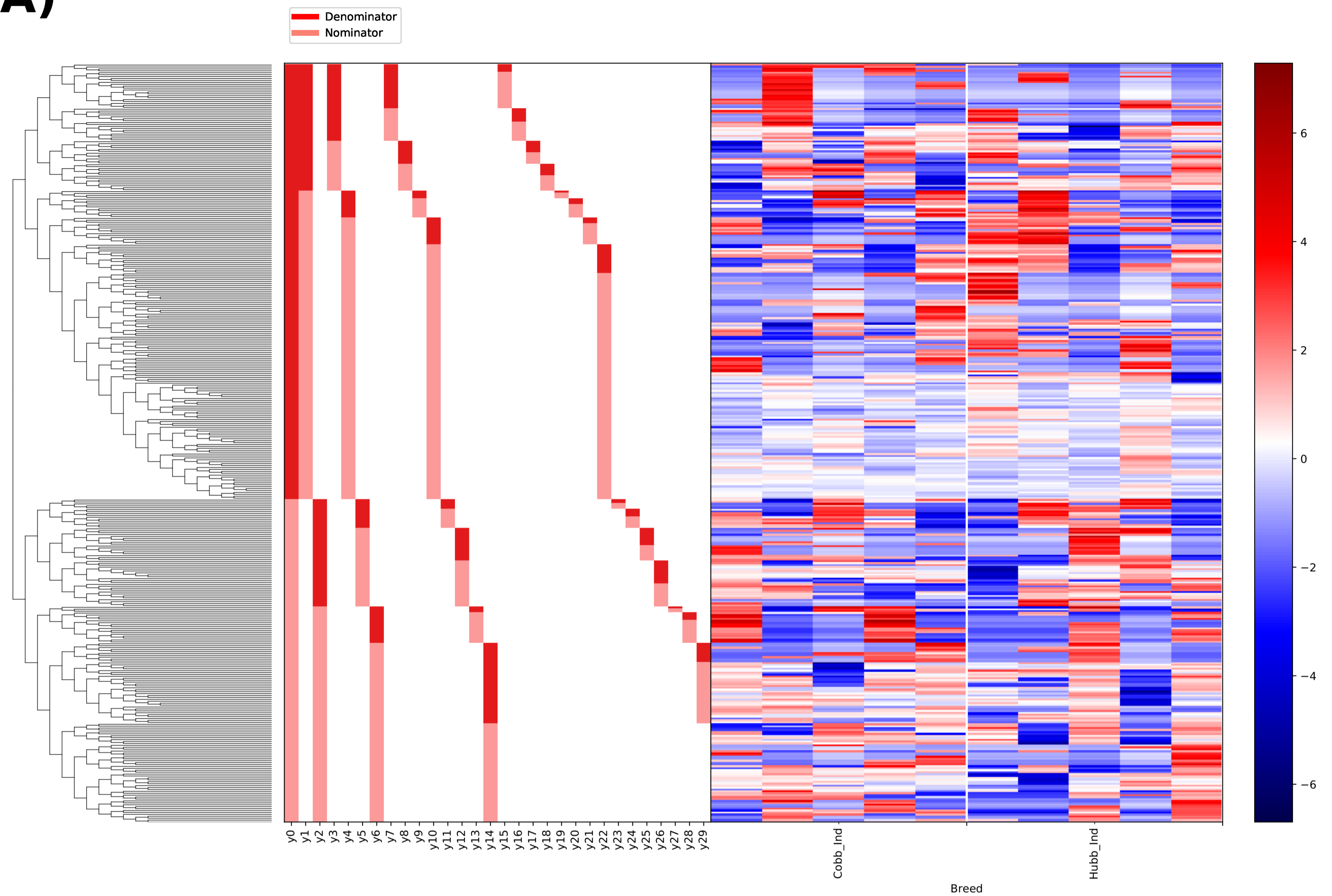

**B)**

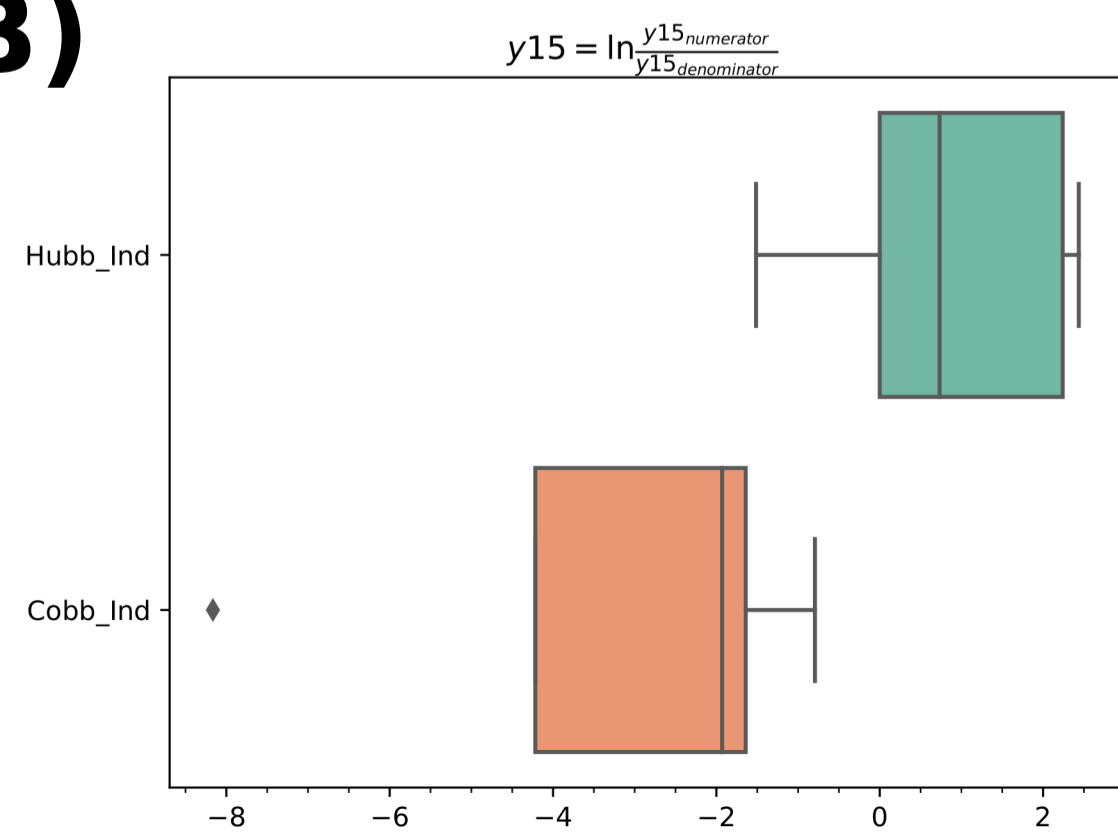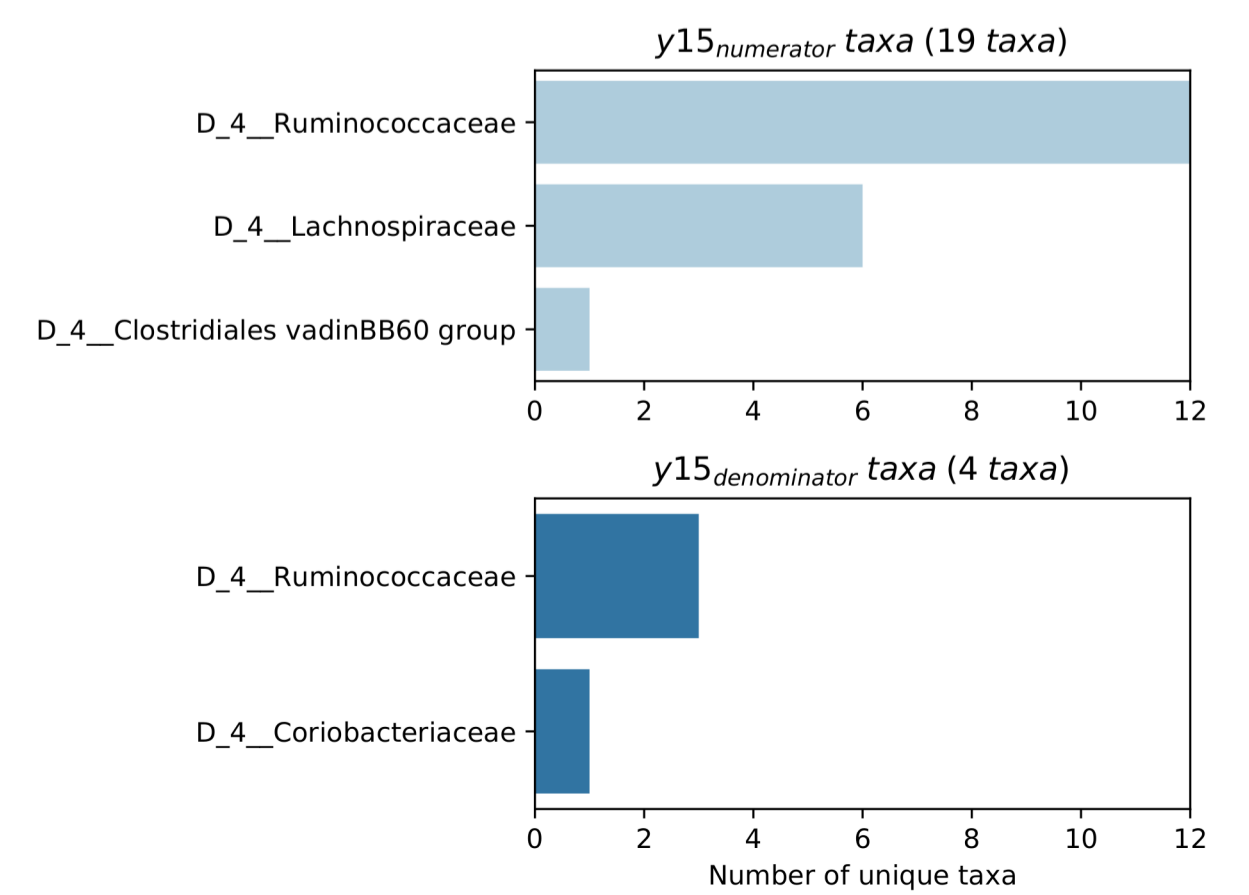

Supplement: Supplementary Figure 5 — A dendrogram heatmap (A) showing differences in the caecal mucus microbiota of Cobb and Hubbard chickens at 42 d.p.h. The log ratio of balance y15 (B) was significantly lower in Cobb chickens due to an increased relative abundance of denominator ASVs. [file Image_5.pdf]
